# Traditional herbal medicine for anorexia in patients with cancer: a systematic review and meta-analysis of randomized controlled trials

**DOI:** 10.3389/fphar.2023.1203137

**Published:** 2023-06-27

**Authors:** Su Bin Park, Jee-Hyun Yoon, Eun Hye Kim, Hayun Jin, Seong Woo Yoon

**Affiliations:** ^1^ Department of Korean Internal Medicine, Kyung Hee University Hospital at Gangdong, Seoul, Republic of Korea; ^2^ Department of Clinical Korean Medicine, Graduate School, Kyung Hee University, Seoul, Republic of Korea

**Keywords:** cancer, anorexia, traditional herbal medicine, systematic review, meta-analysis

## Abstract

**Background:** The purpose of this systematic review and meta-analysis was to evaluate the efficacy and safety of traditional herbal medicine (THM) for improving anorexia in patients with cancer.

**Methods:** We searched for randomized controlled trials (RCTs) that evaluated orally administered THM for cancer-related anorexia using 10 databases from the inception to 1 August 2021. The primary outcome was an improvement in anorexia, measured with the total effective rate (TER) or visual analog scale (VAS). The secondary outcomes were the changes in body weight, the Karnofsky performance scale, acylated ghrelin, and adverse events. We used the Cochrane risk of bias assessment tool and the Grading of Recommendations Assessment, Development, and Evaluation method to assess the quality of the studies and the quality of the evidence.

**Results:** A total of 26 RCTs were included, of which 23 were subjected to quantitative analysis. THM showed a significant improvement in anorexia measured with the TER [risk ratio (RR) 1.12, 95% confidence intervals (CI) 1.04–1.20] than appetite stimulants with moderate quality evidence and in the Karnofsky performance scale (RR 1.38, 95% CI 1.12–1.70) with low quality evidence but not in body weight gain (RR 0.98, 95% CI 0.80–1.20). THM showed a significant improvement in anorexia measured with the TER (RR 1.74, 95% CI 1.23–2.48) compared with usual care with low-quality evidence but did not significantly improve the VAS score (mean difference 0.72, 95% CI 0.00–1.43) or the level of acylated ghrelin (mean difference 0.94, 95% CI 1.08–2.97). There were no serious adverse events.

**Conclusion:** This review suggests that THM may be considered a safe alternative therapeutic option for improving anorexia in patients with cancer. Nonetheless, more rigorous RCTs are needed due to methodological limitations.

**Systematic Review Registration:** https://www.crd.york.ac.uk/prospero, identifier CRD42021276508.

## 1 Introduction

Anorexia, a common cancer-related symptom, is defined as a loss of appetite leading to a reduced food intake (Laviano et al., 2017). It occurs in 50% of patients with new cancer diagnoses and 70% of patients with advanced cancer (Yu, 2015; Zhang et al., 2018) and is often accompanied by cachexia, which causes weight loss, tissue wasting, poor quality of life, low response to cancer treatment, and poor survival rates (Trajkovic-Vidakovic et al., 2012; Tarricone et al., 2016; Laviano et al., 2017).

The multifactorial etiology of anorexia in patients with cancer is not fully understood, but systematic inflammation and abnormal neurohormonal responses due to tumor-induced changes in host metabolism are likely to play a major role. Additionally, cancer-related symptoms such as pain, depression, taste disorders, and anti-cancer therapies can contribute to anorexia in these patients (Wiffen et al., 2014; Ezeoke and Morley, 2015; Mattox, 2017). Pharmacological interventions such as progesterone analogs and corticosteroids are recommended for patients with cancer-related anorexia (Roeland et al., 2020). Megestrol acetate, one of the progesterone analogs, improves appetite and increases body weight. However, it can cause thromboembolic events, edema, dyspnea, and adrenal suppression, and increase the risk of deaths especially in higher doses (Garcia et al., 2013). Medroxyprogesterone acetate has shown similar side effects (Roeland et al., 2020). Corticosteroids also improve appetite and quality of life, but they should only be used for a short period of time due to the toxicities such as gastrointestinal bleeding and proximal myopathy and decreased efficacy during long-term use (Miller et al., 2014; Roeland et al., 2020).

Complementary and alternative medicine (CAM) is commonly used in patients with cancer. From 25% to 80% of patients in western countries have used some form of CAM, and 32.2% of them used herbal medicine (Judson et al., 2017; Alsharif F, 2021). Traditional herbal medicine (THM) in East Asian countries is one of the important components of CAM, which includes traditional Korean medicine, traditional Chinese medicine, and traditional Japanese medicine (Kampo). It has been widely used to enhance the efficacy and manage the side effects of standard cancer treatment and to improve cancer-related symptoms (McQuade et al., 2012; Chung et al., 2016; Yoon et al., 2021). A recent study suggested that THM could be used to enhance the effect of immunotherapy and reduce immune-related adverse events by having an impact on both immunological stimulation and immunological suppression (Zhang and Xiao, 2021).

In East Asian countries, 44.6%–83% of patients with cancer had used THM (Iwase et al., 2012; McQuade et al., 2012). 37.8%–64.3% of physicians prescribed THM to manage cancer-related symptoms, of which 16.9%–36% were prescribed for anorexia and weight loss (Iwase et al., 2012; McQuade et al., 2012; Yoon et al., 2021). Other CAM interventions such as auricular acupuncture, acupuncture, and moxibustion are feasible to improve appetite and maintain weight in patients with cancer, but the evidence is insufficient to make a definitive conclusion (Sun et al., 2020; Liu et al., 2021). Preclinical studies have shown that THM ameliorates cancer-related anorexia and cachexia through anti-inflammation, regulation of the neuroendocrine pathway, and modulation of the ubiquitin-proteasome system or protein synthesis (Park et al., 2019). A systematic review and meta-analysis on cancer cachexia reported that THM and acupuncture improve appetite, cachexia-related symptoms, and quality of life in patients with cancer cachexia while also being safe (Xu et al., 2021). Earlier systematic reviews and meta-analyses have demonstrated the therapeutic potential of THM for improving anorexia in palliative cancer care (Chung et al., 2016; Lee et al., 2017; Chen et al., 2021). However, there are shortcomings. Chung et al. discussed overall cancer and cancer treatment-related symptoms rather than focusing on anorexia and included just two randomized controlled trials (RCTs) (Chung et al., 2016). Also reviewed overall chemotherapy-induced gastrointestinal toxicity and only included colorectal cancer patients (Chen et al., 2021). Lee et al. systematically reviewed RCTs published between 2000 and 2015 focused on cancer-related anorexia, but a meta-analysis was not conducted (Lee et al., 2017). More robust clinical evidence of THM focusing on cancer-related anorexia is needed. Therefore, this systematic review and meta-analysis of randomized controlled trials aimed to evaluate the efficacy and safety of THM for anorexia in patients with cancer.

## 2 Materials and methods

### 2.1 Search strategy

Ten electronic databases including PubMed, Cochrane Library, EMBASE, China National Knowledge Infrastructure (CNKI), Japanese databases (CiNii and JSOM), and Korean databases (KMBASE, KISS, NDSL, and OASIS) from their inception date to 1 August 2021 were searched independently by two investigators (SBP and EHK) without any restriction on publication language, region, or date. The search keywords included cancer, neoplasm, anorexia, cachexia, traditional Korean medicine, traditional Chinese medicine, herbal medicine, and Kampo medicine. The search terms were adjusted for the different databases using a highly sensitive search strategy created by the Cochrane Collaboration. The details of the search strategies are provided in Supplementary Material S1.

The systematic review and meta-analysis were conducted according to the Preferred Reporting Items for Systematic Reviews and Meta-Analysis checklist (Page et al., 2021). The protocol for this study had been registered with the International Prospective Register of Systematic Review (PROSPERO) with the registration number CRD42021276508. Since all materials used in this research are published studies, ethical approval was not required.

### 2.2 Study selection

Two investigators independently assessed the eligibility of citations based on the title and abstract. Relevant studies were obtained as full text and screened against the following inclusion criteria: (1) RCTs (parallel or crossover studies); (2) studies involving adult patients (age ≥18 years); (3) studies of patients with cancer who have anorexia, cachexia, or both; (4) studies that used oral administration of THM as an interventional group; and (5) studies that used conventional treatment, appetite stimulants, usual care or routine care, a placebo, or no treatment as a control group. Studies were excluded if they met any of the following criteria: (1) studies on THM administered intravenously or externally; (2) studies that were irrelevant to anorexia; and (3) studies that involved other interventions such as acupuncture and moxibustion. Disagreements were resolved through discussion between the reviewers or arbitrated by a third investigator (SWY) if necessary.

### 2.3 Outcome measures

The primary outcome was to assess the improvement in clinical symptoms of anorexia after the intervention, as measured by changes in food intake or anorexia score using tools such as the total effective rate (TER) or visual analog scale (VAS). The TER is calculated as the percentage of participants who were cured, markedly effective, or improved out of the total number enrolled. The secondary outcomes included the change in body weight, the quality of life measured using the Karnofsky performance scale, the plasma level of acylated ghrelin, and any adverse events that occurred during the treatment period.

### 2.4 Data extraction

Two investigators independently assessed the literature and extracted data using a standardized form. The following data were recorded: name of the first author, publication year, sample size, patient characteristics, cancer type, cancer stage, intervention, comparison, outcomes, and adverse events. Disagreements were resolved through discussion or by consulting a third investigator. If any data were missing, the corresponding author of that respective study was contacted if contact information was available.

### 2.5 Quality assessment

The Cochrane risk of bias tool from the Cochrane handbook version 5.2 was used to evaluate the methodological quality of the included RCTs across seven domains: random sequence generation, allocation concealment, blinding of participants and personnel, blinding of outcome assessment, incomplete outcome, selective reporting, and other bias (Higgins et al., 2017). Each domain was assessed as having a low (“L”), uncertain (“U”), or high (“H”) risk of bias. If baseline characteristics were different, other bias was assessed as high risk. Two investigators independently conducted the risk of bias assessment, and disagreements were resolved by consulting a third investigator.

### 2.6 Statistical analysis

Statistical analyses were performed by the RevMan software (Version 5.4, Copenhagen: The Nordic Cochrane Centre, The Cochrane Collaboration, 2014). The mean difference (MD) with 95% confidence intervals (CI) was used for continuous outcomes and the risk ratios (RR) with 95% CI for dichotomous outcomes (Higgins et al., 2017). If less than five studies were included in the comparison and the outcome measurements were consistent across all of the studies, a fixed-effects model was used; otherwise, a random-effects model was used (Tufanaru et al., 2015). The Cochrane chi-square test, with a significance threshold set at 0.10, and the *I* ^2^ statistic were used to measure the heterogeneity between studies. A value of *I* ^2^ > 50% suggests significant heterogeneity. A funnel plot was used to examine any potential publication bias if there were more than 10 trials in a single outcome.

The quality of the evidence for the results was evaluated using the Grading of Recommendations Assessment, Development, and Evaluation (GRADE) system. Based on the risk of bias, inconsistency, indirectness, impression, and publication bias, each outcome was classified as “high,” “moderate,” “low,” or “very low” quality (Schünemann et al., 2013).

## 3 Results

### 3.1 Selection

**FIGURE 1:**
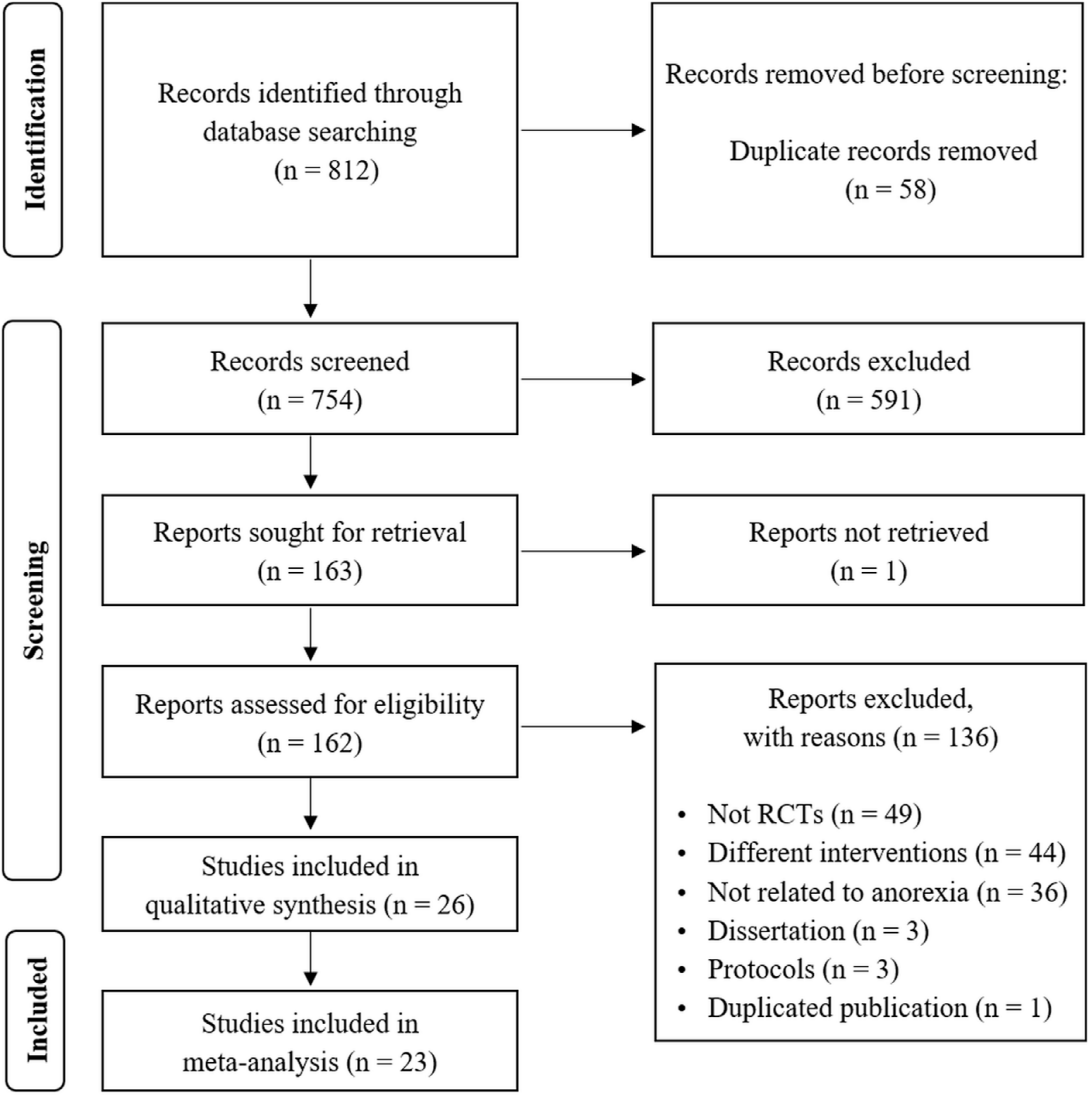
PRISMA flowchart of study selection. PRISMA, Preferred Reporting Items for Systematic Reviews and Meta-Analysis.

The search of electronic databases identified 812 studies, of which 58 duplicates were removed. After screening titles and abstracts, 591 studies were excluded, and one study was excluded because the full text was unavailable. The remaining 162 full-text articles were assessed for eligibility, and 136 studies were excluded for the following reasons: not RCTs (*n* = 50); different interventions (*n* = 45); not related to anorexia (*n* = 37); protocol studies (*n* = 3); and duplicated publication (*n* = 1). Finally, a total of 26 studies were included in the systematic review (Deng, 1997; Zhang, 2000; Cai et al., 2003; Chen, 2007; He et al., 2007; Hu et al., 2007; Kuang et al., 2009; Li and Li, 2011; Ohno et al., 2011; Yan et al., 2012; Wang et al., 2013a; Wang et al., 2013b; Qiu and Zhang, 2013; Li et al., 2014; Zhang et al., 2014; Cui et al., 2015; Huang et al., 2015; Yu, 2015; Yi, 2016; Cheon et al., 2017; Ohnishi et al., 2017; Wang et al., 2018; Hamai et al., 2019; Yoshiya et al., 2020; Zhang and Wang, 2020; Ko M. H. et al., 2021). A meta-analysis was conducted using the data from 23 studies (Deng, 1997; Zhang, 2000; Cai et al., 2003; Chen, 2007; He et al., 2007; Hu et al., 2007; Kuang et al., 2009; Li and Li, 2011; Ohno et al., 2011; Yan et al., 2012; Wang et al., 2013a; Wang et al., 2013b; Qiu and Zhang, 2013; Li et al., 2014; Zhang et al., 2014; Cui et al., 2015; Yu, 2015; Yi, 2016; Ohnishi et al., 2017; Wang et al., 2018; Hamai et al., 2019; Yoshiya et al., 2020; Ko S. J. et al., 2021). The detailed study selection process is shown in Figure 1.

### 3.2 Study characteristics

The characteristics of the included studies are summarized in Table 1. Twenty-six RCTs were published from 1997 to 2021, with most studies conducted in China (*n* = 20) (Deng, 1997; Zhang, 2000; Cai et al., 2003; Chen, 2007; He et al., 2007; Hu et al., 2007; Kuang et al., 2009; Li and Li, 2011; Yan et al., 2012; Wang et al., 2013a; Wang et al., 2013b; Qiu and Zhang, 2013; Li et al., 2014; Zhang et al., 2014; Cui et al., 2015; Huang et al., 2015; Yu, 2015; Yi, 2016; Wang et al., 2018; Zhang and Wang, 2020), followed by Japan (*n* = 4) (Ohno et al., 2011; Ohnishi et al., 2017; Hamai et al., 2019; Yoshiya et al., 2020), and Korea (*n* = 2) (Cheon et al., 2017; Ko M. H. et al., 2021). The sample size varied from 10 to 195, with a mean patient age ranging from 43.1 to 69 years old. Seventeen RCTs included various cancer types (Zhang, 2000; Cai et al., 2003; He et al., 2007; Li and Li, 2011; Yan et al., 2012; Wang et al., 2013a; Wang et al., 2013b; Qiu and Zhang, 2013; Li et al., 2014; Zhang et al., 2014; Cui et al., 2015; Huang et al., 2015; Yu, 2015; Cheon et al., 2017; Wang et al., 2018; Zhang and Wang, 2020; Ko S. J. et al., 2021), and three studies did not specify the cancer type (Deng, 1997; Chen, 2007; Yi, 2016). Other studies included specific cancer types such as lung (He et al., 2007; Kuang et al., 2009; Yoshiya et al., 2020), esophageal (Hamai et al., 2019), gastric (Ohno et al., 2011), and cervical (Ohnishi et al., 2017) cancers. Fifteen RCTs did not mention the cancer stage (Deng, 1997; Cai et al., 2003; He et al., 2007; Li and Li, 2011; Wang et al., 2013b; Qiu and Zhang, 2013; Huang et al., 2015; Yu, 2015; Yi, 2016; Cheon et al., 2017; Ohnishi et al., 2017; Wang et al., 2018; Yoshiya et al., 2020; Zhang and Wang, 2020; Ko M. H. et al., 2021), while the remaining 10 studies enrolled patients with cancer stages III or IV (Chen, 2007; Hu et al., 2007; Kuang et al., 2009; Ohno et al., 2011; Yan et al., 2012; Wang et al., 2013a; Li et al., 2014; Zhang et al., 2014; Cui et al., 2015; Zhang and Wang, 2020). Eight studies enrolled patients undergoing chemotherapy (He et al., 2007; Ohno et al., 2011; Qiu and Zhang, 2013; Huang et al., 2015; Ohnishi et al., 2017; Hamai et al., 2019; Yoshiya et al., 2020) or radiotherapy (Deng, 1997), and eleven studies included patients diagnosed with cancer and cachexia-anorexia syndrome (Zhang, 2000; Cai et al., 2003; Chen, 2007; Hu et al., 2007; Kuang et al., 2009; Li and Li, 2011; Yan et al., 2012; Wang et al., 2013a; Zhang et al., 2014; Yu, 2015; Wang et al., 2018).

**TABLE 1:** Basic characteristics of included studies.

Study id	N	Mean age (I/C)	Cancer type	Stage	Cause	Intervention	Control	Duration	Outcome
Cui et al. (2015)	120	(52.9/53.8)	Breast, lung, stomach, etc.	III, IV	N/A	Kaiwei Jinshi Tang	MGA	2 weeks	FA NRS, KPS, BW
Hu et al. (2007)	60	59.3	Lung	III, IV	CAS	Shenqijiaocao Dec	MGA	4 weeks	KPS, PI symptoms, FA, BW, AL
Huang et al. (2015)	60	(58.50/59.26)	Lung, breast, etc.	N/A	CTX	Xiangsha Liujunzi Dec	MPA	2 weeks	AG, FA
Kuang et al. (2009)	120	60.2	Lung	III, IV	CAS	Yiqi Yangyin Dec	MGA	4 weeks	KPS, PI symptoms, FA, BW, AL
Li and Li (2011)	80	(57.00/57.23)	Lung, stomach, etc.	N/A	CAS	Jianpi Huatan Dec	MGA	4 weeks	SY, FA, KPS, AL
Li et al. (2014)	115	(66.40/65.48)	Lung, stomach, etc.	III, IV	N/A	Jiaweizhizhu particles	MGA	4 weeks	NRS, FA, KPS
Wang et al. (2013b)	120	(53.2/52.4)	Breast, lung, etc.	N/A	N/A	Shuyu Pill	MGA	3 weeks	AG, FA, KPS, BW
Wang et al. (2018)	90	(63/59)	Lung, stomach, etc.	N/A	CAS	Xiangsha Liujun Dec	MPA	8 weeks	KPS, QOL, FA, survival
Yu (2015)	24	51.0	Esophageal, stomach, etc.	N/A	CAS	Jianpi Huatan Dec	MGA	4 weeks	SY, FA, KPS, AL
Zhang (2000)	52	(60/59)	Stomach, lung, etc.	III, IV	N/A	Fuzheng Peiben Dec	MGA	4 weeks	FA, BW, KPS, WBC, Hb
Yi (2016)	100	50.59	N/A	N/A	N/A	Kaiwei Jinshi Tang	MGA	2 weeks	FA, KPS
Zhang et al. (2014)	75	(57.16/56.80)	Lung, stomach, etc.	III, IV	CAS	Xiaoyan Dec	MGA	8 weeks	FA, KPS, BW, AL, leptin, T lymphocyte, NK cell
Zhang and Wang (2020)	60	(60.27/63.43)	Stomach, colorectal, etc.	N/A	CAS	Fuzi Lizhong Dec	MPA	4 weeks	CTCAE, KPS, BW
Cai et al. (2003)	90	46	Nasopharyngeal, lung, etc.	N/A	CAS	Buzhong Yiqi Tang	MPA	4 weeks	FA, BW
Chen (2007)	56	N/A	N/A	IV	CAS	Zhipu Liujunzi Dec	MGA	3 weeks	FA, BW, QOL
Deng (1997)	195	(53.7/48.1)	N/A	N/A	RTX	Xiangsha Erya Kaiwei Dec	UC	10 days	FA, SY
Hamai et al. (2019)	18	62.9	Esophageal	IB ∼ IV	CTX	Rikkunshito	UC	2 weeks	1. Calorie intake
									2. VAS, ghrelin
He et al. (2007)	66	N/A	Lung, esophagus, etc.	N/A	CTX	Bazhen granule	UC	N/A	FA
Ohnishi et al. (2017)	39	(51.5/43.1)	Uterine	N/A	CTX	Rikkunshito	UC	2 weeks	1. SY
									2. SY, VAS, QOL, ACS of FAACT, ghrelin
Ohno et al. (2011)	10	61.8	Stomach	III, IV	CTX	Rikkunshito	UC	3 weeks	1. FA, AG, CTCAE
									2. Ghrelin
Qiu and Zhang (2013)	152	(48.2/47.9)	Colorectal, stomach, etc.	N/A	CTX	Shenling Baizhu san	UC	1 weeks	1. AG
									2. Bone marrow suppression
Wang et al. (2013a)	120	(52.9/52.5)	Lung, breast, etc.	IV	CAS	Shuyu Pill	UC	3 weeks	FA, KPS, BW
Yan et al. (2012)	123	(60.8/60.1)	Colorectal, lung, etc.	IV	CAS	Xiangsha Zhizhu Jiawe Dec	UC	2 weeks	FA, BW
Yoshiya et al. (2020)	39	(69/67)	Lung	N/A	CTX	Rikkunshito	UC	2 weeks	1. Caloric intake
									2. Ghrelin, FLIE
Ko et al. (2021a)	40	(51.5/47.55)	Solid	N/A	N/A	Yukgunja-tang	UC	4 weeks	1. ACS of FAACT
									2. FAACT except ACS, VAS, laboratory test
Cheon et al. (2017)	32	(54.1/55.1)	Thyroid, breast, etc.	N/A	N/A	Sipjeondaebo-tang	Placebo	4 weeks	1. ACS of FAACT
									2. FAACT, VAS, laboratory test

ACS, anorexia/cachexia subscale; AG, anorexia grade; AL, albumin; BW, body weight; C, control; CAS, cachexia anorexia syndrome; CTCAE, common terminology criteria for adverse events; CTX, chemotherapy; Dec, decoction; FA, food amount; FAACT, functional assessment of anorexia-cachexia therapy; FLIE, functional living index-emesis; I, intervention; KPS, karnofsky performance score; MGA, megestrol acetate; MPA, medroxyprogesterone acetate; N/A, not available; NRS, numeric rating scale; PI, pattern identification; QOL, quality of life; RTX, radiotherapy; SY, symptom; UC, usual care; VAS, visual analog scale.

Eight studies included patients with anorexia correlated with particular pattern identification (Hu et al., 2007; Kuang et al., 2009; Qiu and Zhang, 2013; Li et al., 2014; Cui et al., 2015; Cheon et al., 2017; Wang et al., 2018; Zhang and Wang, 2020). Spleen-stomach deficiency (*n* = 5) (Qiu and Zhang, 2013; Li et al., 2014; Cui et al., 2015; Wang et al., 2018; Zhang and Wang, 2020), Qi-blood deficiency (*n* = 2) (Hu et al., 2007; Cheon et al., 2017), and Qi-yin deficiency (*n* = 1) (Kuang et al., 2009) were the three pattern identification categories (not shown in Table 1). For Spleen-stomach deficiency, Jiaweizhizhu particles (Li et al., 2014), Kaiwei Jinshi Tang (Cui et al., 2015), Xiangsha Liujun decoction (Wang et al., 2018), Fuzi Lizhong decoction (Zhang and Wang, 2020), and Shenling Baizhu San (Qiu and Zhang, 2013) were prescribed. Sipjeondaebo-Tang (Cheon et al., 2017) and Shenqijiaocao decoction (Hu et al., 2007) were used to treat Qi-blood deficiency, and Yiqi Yangyin decoction (Kuang et al., 2009) was used to treat Qi-yin deficiency.

Various types of herbal formulas were used in the included RCTs, with Yukgunja-tang (YGJT) (Rikkunshito in Japanese or Liujunzi decoction in Chinese) (Ohno et al., 2011; Ohnishi et al., 2017; Hamai et al., 2019; Yoshiya et al., 2020; Ko S. J. et al., 2021) or modified YGJT (Chen, 2007; Huang et al., 2015; Wang et al., 2018) being the most frequently used THM. *Citrus aurantium* L. (*C. aurantium*) was the most frequently used herb in the included RCTs and the details of THM prescriptions and the frequency of herbs are shown in Supplementary Materials S2–S4.

The control group was treated with appetite stimulants such as megestrol acetate (Zhang, 2000; Chen, 2007; Hu et al., 2007; Kuang et al., 2009; Li and Li, 2011; Wang et al., 2013b; Li et al., 2014; Zhang et al., 2014; Cui et al., 2015; Yu, 2015; Yi, 2016) and medroxyprogesterone (Cai et al., 2003; Huang et al., 2015; Wang et al., 2018; Zhang and Wang, 2020), or usual care such as digestive medicine (Deng, 1997; He et al., 2007; Yan et al., 2012), antiemetics (Ohno et al., 2011; Qiu and Zhang, 2013; Ohnishi et al., 2017; Hamai et al., 2019; Yoshiya et al., 2020), parenteral nutrition (He et al., 2007), nutritional counseling (Ko M. H. et al., 2021), the best supportive care (Wang et al., 2013a), or a placebo (Cheon et al., 2017).

The primary outcome was reported with the TER of improvement in anorexia (Deng, 1997; Zhang, 2000; Cai et al., 2003; Chen, 2007; He et al., 2007; Hu et al., 2007; Kuang et al., 2009; Li and Li, 2011; Yan et al., 2012; Wang et al., 2013a; Wang et al., 2013b; Qiu and Zhang, 2013; Li et al., 2014; Zhang et al., 2014; Cui et al., 2015; Yu, 2015; Yi, 2016; Wang et al., 2018), the VAS score of anorexia (Cheon et al., 2017; Ohnishi et al., 2017; Hamai et al., 2019; Ko S. J. et al., 2021), or other measures (Ohno et al., 2011; Huang et al., 2015; Yoshiya et al., 2020; Zhang and Wang, 2020). The secondary outcomes were reported with the TER of improvement in the Karnofsky performance scale (Zhang, 2000; Hu et al., 2007; Kuang et al., 2009; Wang et al., 2013a; Wang et al., 2013b; Li et al., 2014; Zhang et al., 2014; Cui et al., 2015; Yi, 2016), the TER of body weight gain (Zhang, 2000; Cai et al., 2003; Chen, 2007; Hu et al., 2007; Kuang et al., 2009; Yan et al., 2012; Wang et al., 2013b; Zhang et al., 2014), the level of acylated ghrelin (Ohno et al., 2011; Ohnishi et al., 2017; Hamai et al., 2019; Yoshiya et al., 2020), or other measures (Li and Li, 2011; Wang et al., 2013a; Cui et al., 2015; Yu, 2015; Zhang and Wang, 2020).

### 3.3 Risk of bias in the included studies

**FIGURE 2:**
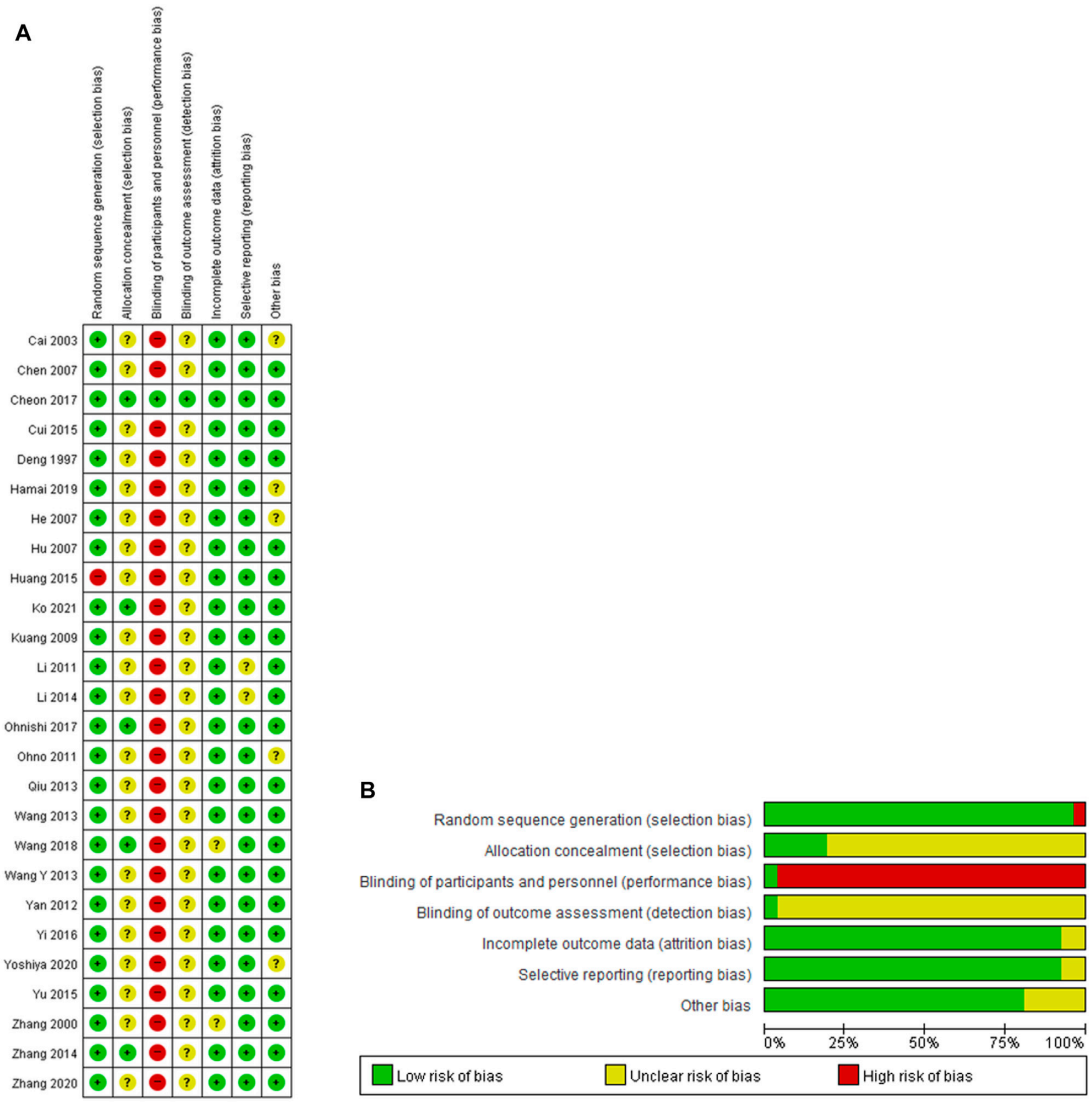
**(A)** Risk of bias summary. **(B)** Risk of bias graph. +, low risk of bias; ?, unclear of bias; -, high risk of bias.

The risk of bias in the included studies is shown in Figure 2. Most studies properly described random sequence generation, although one study had a high selection bias (Huang et al., 2015). In terms of allocation concealment, five studies reported a detailed allocation procedure (Zhang et al., 2014; Cheon et al., 2017; Ohnishi et al., 2017; Wang et al., 2018; Ko M. H. et al., 2021), while the remaining 21 studies had an unclear bias (Deng, 1997; Zhang, 2000; Cai et al., 2003; Chen, 2007; He et al., 2007; Hu et al., 2007; Kuang et al., 2009; Li and Li, 2011; Ohno et al., 2011; Yan et al., 2012; Wang et al., 2013a; Wang et al., 2013b; Qiu and Zhang, 2013; Li et al., 2014; Cui et al., 2015; Huang et al., 2015; Yu, 2015; Yi, 2016; Hamai et al., 2019; Yoshiya et al., 2020; Zhang and Wang, 2020). Blinding of participants and personnel was not performed in 25 studies (Deng, 1997; Zhang, 2000; Cai et al., 2003; Chen, 2007; He et al., 2007; Hu et al., 2007; Kuang et al., 2009; Li and Li, 2011; Ohno et al., 2011; Yan et al., 2012; Wang et al., 2013a; Wang et al., 2013b; Qiu and Zhang, 2013; Li et al., 2014; Zhang et al., 2014; Cui et al., 2015; Huang et al., 2015; Yu, 2015; Yi, 2016; Ohnishi et al., 2017; Wang et al., 2018; Hamai et al., 2019; Yoshiya et al., 2020; Zhang and Wang, 2020; Ko S. J. et al., 2021), but one study using a placebo was graded as having low-performance bias (Cheon et al., 2017). Blinding of outcome assessment was presented in one study (Cheon et al., 2017), whereas other studies with no such data were determined to be unclear (Deng, 1997; Zhang, 2000; Cai et al., 2003; Chen, 2007; He et al., 2007; Hu et al., 2007; Kuang et al., 2009; Li and Li, 2011; Ohno et al., 2011; Yan et al., 2012; Wang et al., 2013a; Wang et al., 2013b; Qiu and Zhang, 2013; Li et al., 2014; Zhang et al., 2014; Cui et al., 2015; Huang et al., 2015; Yu, 2015; Yi, 2016; Ohnishi et al., 2017; Wang et al., 2018; Hamai et al., 2019; Yoshiya et al., 2020; Zhang and Wang, 2020; Ko M. H. et al., 2021). In terms of incomplete outcome data, two studies were unclear (Zhang, 2000; Wang et al., 2018), and the other studies were assessed as having a low risk of bias (Deng, 1997; Cai et al., 2003; Chen, 2007; He et al., 2007; Hu et al., 2007; Kuang et al., 2009; Li and Li, 2011; Ohno et al., 2011; Yan et al., 2012; Wang et al., 2013a; Wang et al., 2013b; Qiu and Zhang, 2013; Li et al., 2014; Zhang et al., 2014; Cui et al., 2015; Huang et al., 2015; Yu, 2015; Yi, 2016; Cheon et al., 2017; Ohnishi et al., 2017; Hamai et al., 2019; Yoshiya et al., 2020; Zhang and Wang, 2020; Ko S. J. et al., 2021). Two studies were unclear in reporting bias (Li and Li, 2011; Li et al., 2014), whereas the other 24 studies were evaluated as low risk (Deng, 1997; Zhang, 2000; Cai et al., 2003; Chen, 2007; He et al., 2007; Hu et al., 2007; Kuang et al., 2009; Ohno et al., 2011; Yan et al., 2012; Wang et al., 2013a; Wang et al., 2013b; Qiu and Zhang, 2013; Zhang et al., 2014; Cui et al., 2015; Huang et al., 2015; Yu, 2015; Yi, 2016; Cheon et al., 2017; Ohnishi et al., 2017; Wang et al., 2018; Hamai et al., 2019; Yoshiya et al., 2020; Zhang and Wang, 2020; Ko M. H. et al., 2021). Except for five studies (Cai et al., 2003; He et al., 2007; Ohno et al., 2011; Hamai et al., 2019; Yoshiya et al., 2020), the other 21 studies were considered to have a low risk of other biases because there were no differences in baseline between the intervention and control groups (Deng, 1997; Zhang, 2000; Chen, 2007; Hu et al., 2007; Kuang et al., 2009; Li and Li, 2011; Yan et al., 2012; Wang et al., 2013a; Wang et al., 2013b; Qiu and Zhang, 2013; Li et al., 2014; Zhang et al., 2014; Cui et al., 2015; Huang et al., 2015; Yu, 2015; Yi, 2016; Cheon et al., 2017; Ohnishi et al., 2017; Wang et al., 2018; Zhang and Wang, 2020; Ko M. H. et al., 2021).

### 3.4 THM versus appetite stimulants

Fifteen studies compared THM with appetite stimulants (Zhang, 2000; Cai et al., 2003; Chen, 2007; Hu et al., 2007; Kuang et al., 2009; Li and Li, 2011; Wang et al., 2013b; Li et al., 2014; Zhang et al., 2014; Cui et al., 2015; Huang et al., 2015; Yu, 2015; Yi, 2016; Wang et al., 2018; Zhang and Wang, 2020), and the treatment duration of THM ranged from two to 8 weeks. Eleven studies included various cancer types (Zhang, 2000; Cai et al., 2003; Li and Li, 2011; Wang et al., 2013b; Li et al., 2014; Zhang et al., 2014; Cui et al., 2015; Huang et al., 2015; Yu, 2015; Wang et al., 2018; Zhang and Wang, 2020); two included lung cancer (Hu et al., 2007; Kuang et al., 2009); and the remaining two did not indicate the cancer type (Chen, 2007; Yi, 2016). Anorexia was induced by cachexia-anorexia syndrome in nine studies (Cai et al., 2003; Chen, 2007; Hu et al., 2007; Kuang et al., 2009; Li and Li, 2011; Zhang et al., 2014; Yu, 2015; Wang et al., 2018; Zhang and Wang, 2020), chemotherapy in one study (Huang et al., 2015), and an unknown etiology in five studies (Zhang, 2000; Wang et al., 2013b; Li et al., 2014; Cui et al., 2015; Yi, 2016).

#### 3.4.1 Anorexia

**FIGURE 3:**
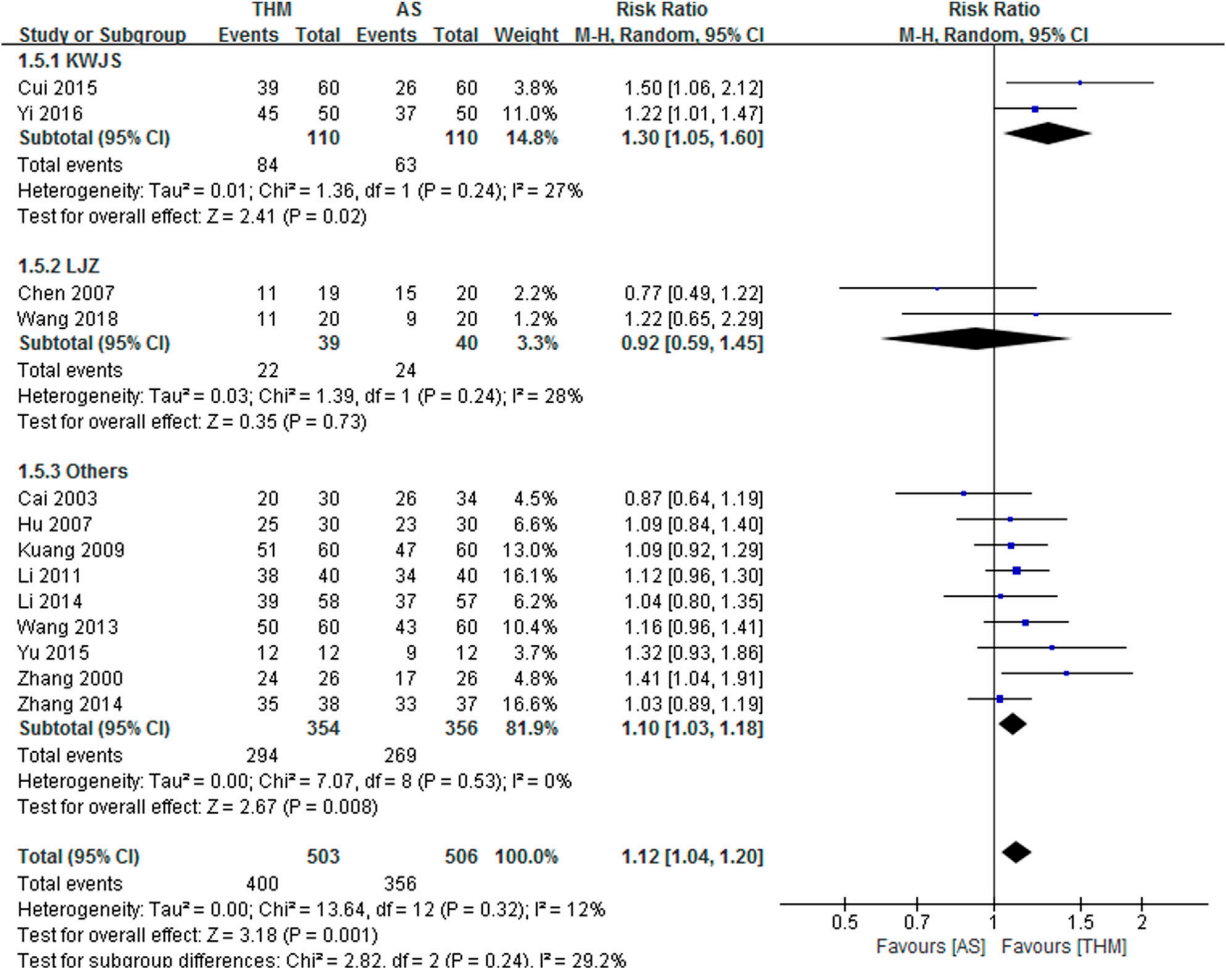
Forest plot of the total effective rate of THM versus AS for anorexia. THM, traditional herbal medicine; AS, appetite stimulants; LJZ, Liujunzi decoction; KWJS, kaiweijinshi tang; CI, confidence intervals.

Thirteen RCTs with a total of 1,009 patients reported the TER of THM versus appetite stimulants for improving anorexia and were included in the meta-analysis (Figure 3) (Zhang, 2000; Cai et al., 2003; Chen, 2007; Hu et al., 2007; Kuang et al., 2009; Li and Li, 2011; Wang et al., 2013b; Li et al., 2014; Zhang et al., 2014; Cui et al., 2015; Yu, 2015; Yi, 2016; Wang et al., 2018). Overall, THM showed a statistically significant improvement in anorexia compared to appetite stimulants (RR 1.12, 95% CI 1.04 to 1.20, *p* = 0.001) with a low grade of heterogeneity (*I* ^*2*^ = 12%). In subgroup analysis according to herbal formula, Kaiwei Jinshi Tang (Cui et al., 2015; Yi, 2016) showed significant improvement in anorexia compared to appetite stimulants (RR 1.30, 95% CI 1.05 to 1.60, *p* = 0.02), whereas Liujunzi decoction (Chen, 2007; Wang et al., 2018) did not (RR 0.92, 95% CI 0.59 to 1.45, *p* = 0.73). The remaining THM (Zhang, 2000; Cai et al., 2003; Hu et al., 2007; Kuang et al., 2009; Li and Li, 2011; Wang et al., 2013b; Li et al., 2014; Zhang et al., 2014; Yu, 2015) showed a significant improvement in anorexia compared to appetite stimulants (RR 1.10, 95% CI 1.03 to 1.18, *p* = 0.008). Based on the GRADE profile, the TER of THM compared to appetite stimulants has a moderate quality of evidence due to methodological limitations, as shown in Table 2.

**TABLE 2:** Summary of findings.

THM compared to appetite stimulants or usual care for anorexia in patients with cancer					
**Patient or population:** Anorexia in patients with cancer					
**Intervention:** THM					
**Comparison:** Appetite stimulants					
**Outcomes**	**Anticipated absolute effects** ^a^ **(95% CI)**		**Relative effect (95% CI)**	**No. Of participants (studies)**	**Certainty of the evidence (GRADE)**
	**Risk with appetite stimulants**	**Risk with THM**			
**Anorexia (TER)**	704 per 1,000	788 per 1,000 (732–844)	RR 1.12 (1.04–1.20)	1,009 (13 RCTs)	⊕⊕⊕○
					Moderate
**Body Weight (TER)**	502 per 1,000	492 per 1,000 (401–602)	RR 0.98 (0.80–1.20)	530 (7 RCTs)	⊕⊕○○
					Low
**Karnofsky Performance Scale (TER)**	553 per 1,000	763 per 1,000 (619–939)	RR 1.38 (1.12–1.70)	762 (8 RCTs)	⊕⊕○○
					Low
**Patient or population:** Anorexia in patients with cancer					
**Intervention:** THM					
**Comparison:** Usual care					
**Outcomes**	**Anticipated absolute effects** ^a^ **(95% CI)**		**Relative effect (95% CI)**	**No. Of participants (studies)**	**Certainty of the evidence (GRADE)**
	**Risk with usual care**	**Risk with THM**			
**Anorexia (TER)**	545 per 1,000	949 per 1,000 (671–1,000)	RR 1.74 (1.23–2.48)	656 (5 RCTs)	⊕⊕○○
					Low
**Anorexia (VAS)**	The mean VAS was 0	MD 0.72 higher (0–1.43 higher)	—	94 (3 RCTs)	⊕○○○
					Very low
**Acylated Ghrelin**	The mean acylated ghrelin was 0	MD 0.94 higher (1.08 lower to 2.97 higher)	—	153 (4 RCTs)	⊕⊕○○
					Low

^a^ The risk in the intervention group (and its 95% CI) is based on the assumed risk in the comparison group and the relative effect of the intervention (and its 95% CI).

GRADE, working group grades of evidence.

High certainty: We are very confident that the true effect lies close to that of the estimate of the effect.

Moderate certainty: We are moderately confident in the effect estimate: the true effect is likely to be close to the estimate of the effect, but there is a possibility that it is substantially different.

Low certainty: Our confidence in the effect estimate is limited: the true effect may be substantially different from the estimate of the effect.

Very low certainty: We have very little confidence in the effect estimate: the true effect is likely to be substantially different from the estimate of effect.

CI, confidence interval; MD, mean difference; RR, risk ratio; TER, total effective rate; THM, traditional herbal medicine; VAS, visual analog scale.

#### 3.4.2 Body weight

**FIGURE 4:**
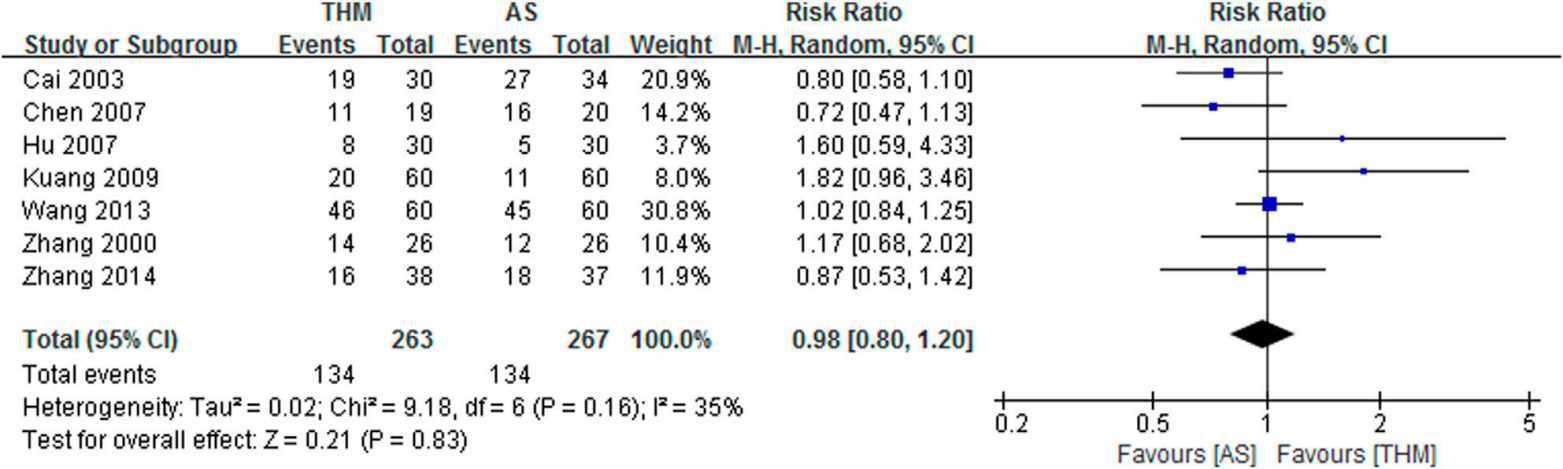
Forest plot of the total effective rate of THM versus AS for body weight. THM, traditional herbal medicine; AS, appetite stimulants; CI, confidence intervals.

Seven RCTs with a total of 530 patients reported the TER of THM versus appetite stimulants for body weight gain and were included in the meta-analysis (Figure 4) (Zhang, 2000; Cai et al., 2003; Chen, 2007; Hu et al., 2007; Kuang et al., 2009; Wang et al., 2013b; Zhang et al., 2014). THM did not show significance in body weight gain compared to appetite stimulants (RR 0.98, 95% CI 0.80 to 1.20, *p* = 0.83) with a low grade of heterogeneity (*I* ^*2*^ = 35%). Based on the GRADE profile, the TER of THM compared to appetite stimulants for body weight gain has a low quality of evidence due to methodological limitations and imprecisions (Table 2).

#### 3.4.3 Karnofsky performance scale

**FIGURE 5:**
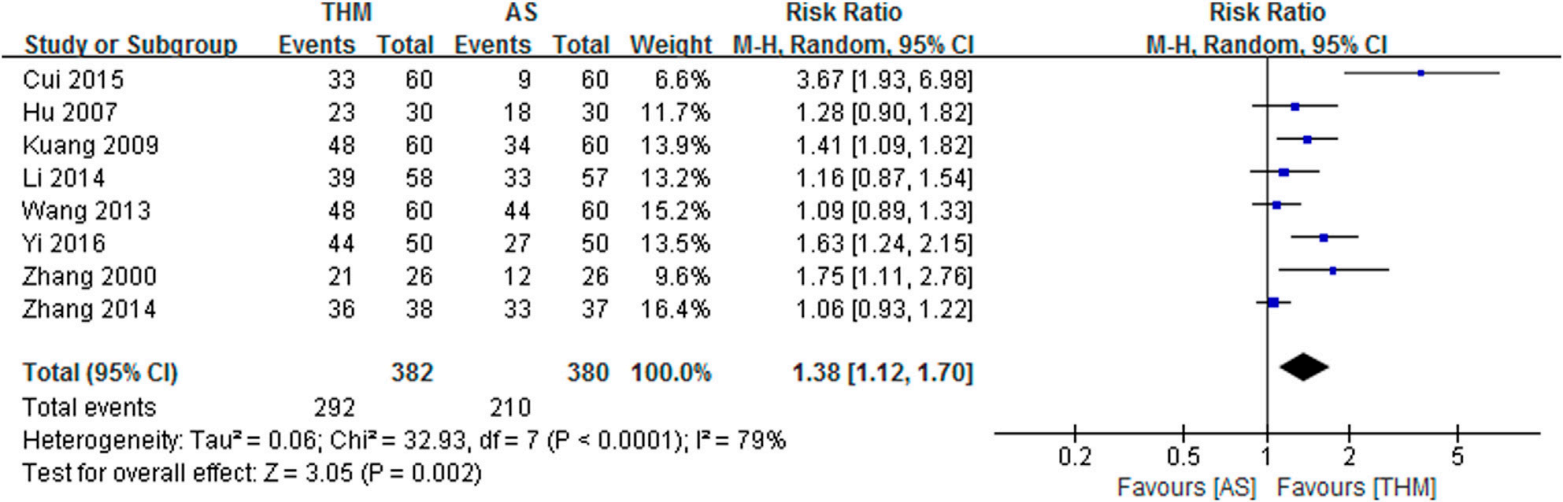
Forest plot of the total effective rate of THM versus AS for the Karnofsky performance scale. THM, traditional herbal medicine; AS, appetite stimulants; CI, confidence intervals.

Eight RCTs involving 762 patients were included in the meta-analysis to compare the TER of THM versus appetite stimulants for improving the Karnofsky performance scale, as depicted in Figure 5 (Zhang, 2000; Hu et al., 2007; Kuang et al., 2009; Wang et al., 2013b; Li et al., 2014; Zhang et al., 2014; Cui et al., 2015; Yi, 2016). The results showed that THM had a statistically significant improvement in the Karnofsky performance scale compared to appetite stimulants (RR 1.38, 95% CI 1.12 to 1.70, *p* = 0.002) with a high grade of heterogeneity (*I* ^*2*^ = 79%). Based on the GRADE profile, the TER of THM versus appetite stimulant for improving the Karnofsky performance scale has a low quality of evidence due to methodological limitations and inconsistencies (Table 2).

### 3.5 THM versus usual care

Ten studies compared THM with usual care (Deng, 1997; He et al., 2007; Ohno et al., 2011; Yan et al., 2012; Wang et al., 2013a; Qiu and Zhang, 2013; Ohnishi et al., 2017; Hamai et al., 2019; Yoshiya et al., 2020; Ko S. J. et al., 2021), with treatment durations of THM ranging from one to 4 weeks. Four of these studies included various cancer types (He et al., 2007; Yan et al., 2012; Wang et al., 2013a; Qiu and Zhang, 2013), and the other four studies included esophageal (Hamai et al., 2019), uterine (Ohnishi et al., 2017), stomach (Ohno et al., 2011), and lung (Yoshiya et al., 2020) cancers, whereas the remaining two studies did not state the cancer type (Deng, 1997; Ko M. H. et al., 2021). Anorexia was induced by chemotherapy in six studies (He et al., 2007; Ohno et al., 2011; Qiu and Zhang, 2013; Ohnishi et al., 2017; Hamai et al., 2019; Yoshiya et al., 2020), cachexia-anorexia syndrome in two studies (Yan et al., 2012; Wang et al., 2013a), radiotherapy in one study (Deng, 1997), and unknown etiology in one study (Ko S. J. et al., 2021).

#### 3.5.1 Anorexia

**FIGURE 6:**
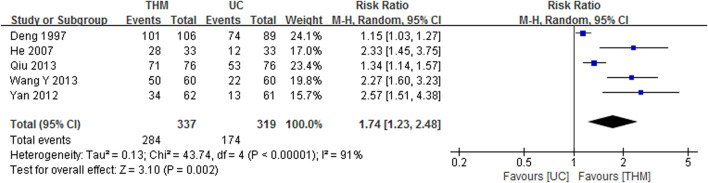
Forest plot of the total effective rate of THM versus UC for anorexia. THM, traditional herbal medicine; UC, usual care; CI, confidence intervals.

Five RCTs with a total of 656 patients were included in the meta-analysis to compare the TER of THM versus usual care for improving anorexia, as shown in Figure 6 (Deng, 1997; He et al., 2007; Yan et al., 2012; Wang et al., 2013a; Qiu and Zhang, 2013). The results showed that THM had a statistically significant improvement in anorexia compared to usual care (RR 1.74, 95% CI 1.23 to 2.48, *p* = 0.002), with a high grade of heterogeneity (*I* ^*2*^ = 91%). Based on the GRADE profile, the TER of THM versus usual care for improving anorexia has a low quality of evidence due to methodological limitations and inconsistencies (Table 2).

**FIGURE 7:**
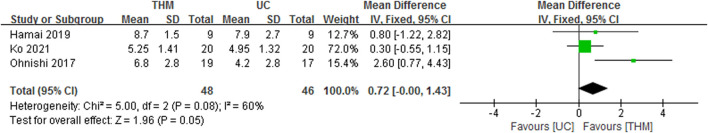
Forest plot of the visual analog scale of THM versus UC for anorexia. THM, traditional herbal medicine; UC, usual care; CI, confidence intervals.

Three RCTs with a total of 94 patients were included in the meta-analysis to compare the VAS score of THM versus usual care for improving anorexia, as depicted in Figure 7 (Ohnishi et al., 2017; Hamai et al., 2019; Ko M. H. et al., 2021). All three included RCTs used YGJT as a THM intervention. The results showed that THM did not have a statistically significant improvement in anorexia compared to usual care (MD 0.72, 95% CI 0.00 to 1.43, *p* = 0.05), with a moderate grade of heterogeneity (*I* ^*2*^ = 60%). However, based on the GRADE profile, the VAS score of THM compared to usual care for improving anorexia has a very low quality of evidence due to methodological limitations, imprecisions, and inconsistencies (Table 2).

#### 3.5.2 Acylated ghrelin

**FIGURE 8:**
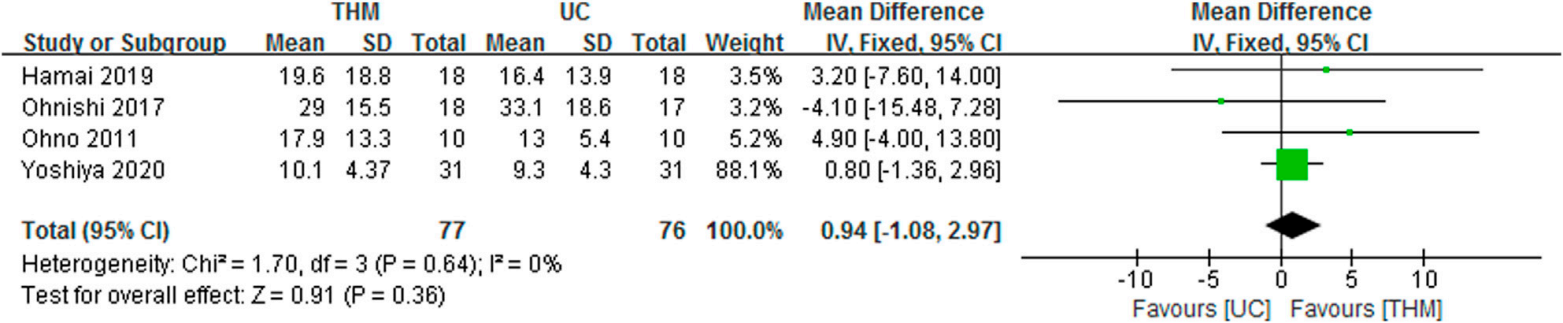
Forest plot of the level of acylated ghrelin in THM versus UC. THM, traditional herbal medicine; UC, usual care; CI, confidence intervals.

Four RCTs with a total of 153 patients reporting the plasma or serum level of acylated ghrelin in THM versus usual care were included in the meta-analysis, as shown in Figure 8 (Ohno et al., 2011; Ohnishi et al., 2017; Hamai et al., 2019; Yoshiya et al., 2020). All of them compared Rikkunshito with usual care for anorexia in patients with cancer treated with cisplatin-based chemotherapy. THM did not show a statistically significantly higher level of acylated ghrelin compared to usual care (MD 0.94, 95% CI 1.08 to 2.97, *p* = 0.36), with a low grade of heterogeneity (*I* ^*2*^ = 0%). However, based on the GRADE profile, the quality of the evidence for the level of acylated ghrelin in THM compared to usual care was low due to methodological limitations and imprecisions (Table 2).

### 3.6 Adverse events

Out of 26 included studies, 13 studies reported adverse events. In 11 out of 13 studies, there were no adverse events related to THM (Chen, 2007; Hu et al., 2007; Ohno et al., 2011; Wang et al., 2013a; Cui et al., 2015; Cheon et al., 2017; Ohnishi et al., 2017; Wang et al., 2018; Hamai et al., 2019; Yoshiya et al., 2020; Zhang and Wang, 2020). Two studies (Zhang, 2000; Ko M. H. et al., 2021) reported mild epigastric bloating, upper respiratory inflammation, and heartburn with no serious THM-related adverse events.

### 3.7 Publication bias

**FIGURE 9:**
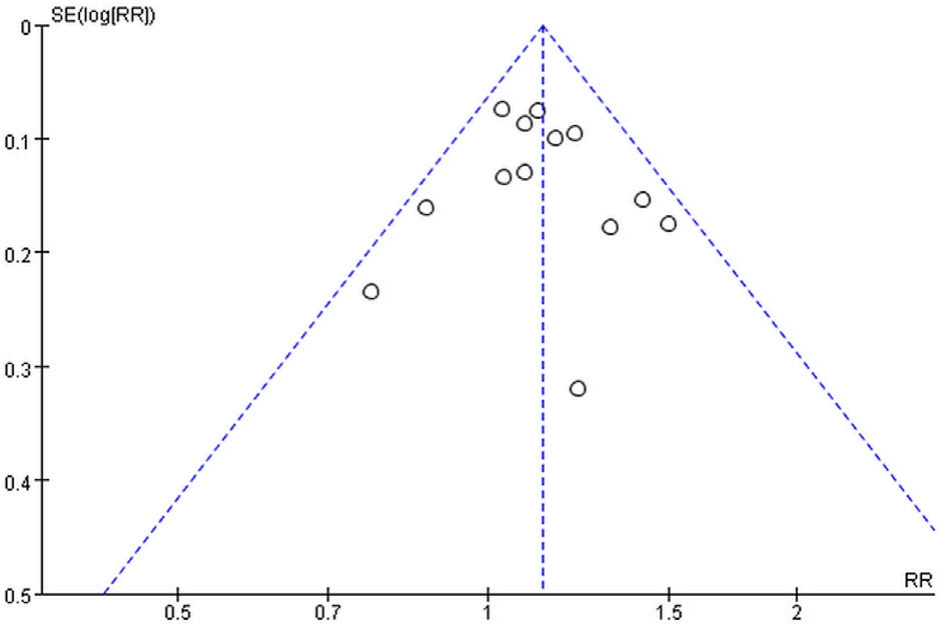
Funnel plot of primary outcome. RR, risk ratio; SE, standard error.

A funnel plot analysis of the 13 RCTs reporting the TER of THM versus appetite stimulants for improvement in anorexia was generated to identify the presence of publication bias (Zhang, 2000; Cai et al., 2003; Chen, 2007; Hu et al., 2007; Kuang et al., 2009; Li and Li, 2011; Wang et al., 2013b; Li et al., 2014; Zhang et al., 2014; Cui et al., 2015; Yu, 2015; Yi, 2016; Wang et al., 2018). Significant asymmetry was not detected, indicating that there was no noticeable publication bias, as shown in Figure 9.

## 4 Discussion

The purpose of this systematic review and meta-analysis was to examine the efficacy and safety of THM in improving anorexia in patients with cancer. This study reviewed 26 studies involving 2,056 patients with cancer suffering from anorexia, of which 23 studies were analyzed quantitatively. The main finding of this study is that THM significantly improved anorexia in patients with cancer compared to appetite stimulants and usual care with moderate and low quality evidence, respectively. Additionally, THM significantly improved the Karnofsky performance scale compared to appetite stimulants, although the quality of the evidence was low. No serious adverse events were reported in the studies evaluating the safety of THM.

Twenty three out of 26 RCTs mentioned the type of cancer, and 3 RCTs did not. There was no relationship between the type of cancer and the THM used to treat cancer-related anorexia. The duration of the THM treatment varies from 1 week to 8 weeks, and 4 weeks was the most common. Although the average treatment period of megestrol acetate and corticosteroids was 8 weeks, the optimal duration of pharmacological treatment remains unknown and long-term use is not recommended (Garcia et al., 2013; Miller et al., 2014; Roeland et al., 2020). Compared to pharmaceutical interventions, the duration of the THM treatment in this study is relatively short, but further research is needed to determine the optimal treatment time because there has been no study comparing the effects of THM depending on the treatment duration.

A previous meta-analysis showed that in children, THM was significantly superior to placebo and active controls such as dietary supplements or conventional medications in improving anorexia symptoms and some biological markers related to appetite (Lee et al., 2022). It also indicated that THM reduces the recurrence rate of anorexia and the incidence of adverse events. Xingpi Yanger granule was the most frequently used herbal formula, and *Crataegus pinnatifida* Bunge, *Atractylodes macrocephala* Koidz, *Poria cocos* (Schw.) Wolf, *C. aurantium*, *Glycyrrhiza uralensis* Fisch. ex DC., and *Hordeum vulgare* L. were frequently used herbs (Lee et al., 2022). These findings are consistent with the results of our study, which also observed that THM improved anorexia in patients with cancer and included commonly used herbs.

In this study, the most frequently used THM prescriptions were YGJT or modified YGJT, which is known as Rikkunshito in Japanese or Liujunzi decoction in Chinese. It is widely used to treat upper gastrointestinal symptoms, including anorexia (Mogami and Hattori, 2014). A previous preclinical study demonstrated that the hesperidin in YGJT improved gastrointestinal motor activities and food intake in rats by inducing endogenous ghrelin secretion through the antagonism of 5-hydroxytryptamine (HT) 2b and 5-HT2c receptors (Fujitsuka et al., 2009). YGJT and its components (10-gingerol) increased plasma levels of acylated ghrelin by inhibiting ghrelin-degrading enzymes in rats treated with cisplatin (Sadakane et al., 2011). YGJT also antagonizes the 5-HT3 receptor (Tominaga et al., 2011). It significantly increased the plasma level of acylated ghrelin in mice and healthy people and upregulated the expression of ghrelin mRNA in the mouse stomach (Matsumura et al., 2010). In clinical studies, YGJT has shown efficacy in improving the symptoms of GERD and functional and drug-associated dyspepsia through its effects on upper gastrointestinal functions and ghrelin secretion signaling. A systematic review and meta-analysis reported that YGJT showed a significantly higher total clinical efficacy rate, higher reduction of total dyspepsia symptom scale, more improved gastric emptying rate and lower recurrence 6 months after treatment in patients with functional dyspepsia compared with western medicine (Ko M. H. et al., 2021). A prior RCT showed that YGJT improved gastrointestinal symptoms and increased plasma ghrelin levels compared to domperidone in patients with FD (Arai et al., 2012). Another RCT demonstrated the efficacy of YGJT against nausea and vomiting caused by selective serotonin reuptake inhibitors (Oka et al., 2007). Although these findings suggest that YGJT has strong evidence for improving anorexia through multiple pathways, our study did not show a statistically significant difference between YGJT and appetite stimulants in treating anorexia or using usual care in the level of acylated ghrelin. These differences may be attributed to concurrent treatment with chemotherapy and a small number of inclusions in the study.

The most frequently used herb in the included RCTs was *C. aurantium*. The dried peel of *C. aurantium* was found to have dual effects by inhibiting the strain of intestinal smooth muscle and increasing gastric emptying and small bowel peristalsis activity (Zhang et al., 2012). It effectively relieves the symptoms induced by reserpine in rats, including poor digestion and absorption capacity, and increases the level of gastrin while lowering motilin and cholecystokinin-8 levels (Zheng et al., 2020). Hesperidin in *C. aurantium* increases the levels of gastrin and decreases the levels of acetylcholine, substance P, motilin, and vasoactive intestinal peptide, while synephrine increases the levels of acetylcholine and motilin and decreases the levels of vasoactive intestinal peptide and substance P (Song et al., 2017). In this study, some RCTs included patients with anorexia who correlated with a specific pattern identification based on the theory of THM (Hu et al., 2007; Kuang et al., 2009; Qiu and Zhang, 2013; Li et al., 2014; Cui et al., 2015; Cheon et al., 2017; Wang et al., 2018; Zhang and Wang, 2020). Most of the pattern identifications were related to deficiency syndromes, including Qi-Blood deficiency, Qi-Yin deficiency, and Spleen-Stomach deficiency. Spleen-Stomach deficiency is a pathological change that leads to a decrease in spleen and stomach activities related to food intake and digestion (World Health Organization Western Pacific Regional Office, 2007). The majority of the herbal formulas in these RCTs have effects that fortify the spleen and promote digestion (Li et al., 2014; Cui et al., 2015), tonify Qi and fortify the spleen (Hu et al., 2007; Qiu and Zhang, 2013; Wang et al., 2018), or tonify Qi and Blood or Yin (Kuang et al., 2009; Cheon et al., 2017). An earlier systematic review reported that invigorating the spleen and regulating Qi therapy is more effective in alleviating symptoms of functional dyspepsia than conventional treatment (Ye et al., 2019). Therefore, it can be assumed that THM, which strengthens the function of the spleen and stomach, encourages digestion, and relieves deficiencies, may be effective for patients with cancer who have anorexia.

Most of the studies that mentioned adverse events reported that there were no adverse events related to THM. Two studies reported mild epigastric bloating, upper respiratory inflammation, and heartburn as THM-related adverse events, and they were not serious (Zhang, 2000; Ko M. H. et al., 2021). Considering that megestrol acetate can cause thromboembolic events, dyspnea, and increased risk of death, and that corticosteroids can be used only for a short period of time due to the adverse events, THM may be considered a safe alternative treatment option (Garcia et al., 2013; Miller et al., 2014; Roeland et al., 2020).

This study has some limitations. First, each study used different outcome measurements, making it difficult to compare and generalize the results. Anorexia was assessed using various scales and indicators, such as the numeric rating scale, the VAS, and food intake. Although food intake and the TER of anorexia were measured, the definitions of efficacy were not consistent across the studies. Second, significant heterogeneity was observed due to differences in treatment duration, doses of appetite stimulants, and the lack of standardization of herbal ingredients. Third, the methodological quality of the included studies was generally limited, with the majority of the studies being graded as having a high or unclear risk of bias due to limited data. In terms of allocation concealment, 21 studies of 26 RCTs did not report the detailed allocation procedure. Blinding of participants and personnel, and blinding of outcome assessment were not performed in 25 studies. As most RCTs in the included studies used positive control such as appetite stimulant or usual care, blinding of participants and personnel could not be performed. Fourth, the long-term effect of THM on cancer-related anorexia could not be determined as most studies had short treatment durations (less than 4 weeks) and did not have a follow-up after treatment. Finally, since the majority of the studies were conducted in Asian countries, it is difficult to generalize these results to broader populations with cancer.

Therefore, large-sample, double-blind RCTs with rigorous methodological evidence are needed. Future studies need to compare the effects of THM depending on the treatment duration to determine the optimal duration. To confirm the long-term effect of THM, follow-up after treatment is also considered. Finally, they should consider a generalized population, standardized herbal ingredients, and standardized outcome measurements to generalize the results to broad populations with cancer.

Nonetheless, this systematic review and meta-analysis is the first attempt to focus on the potential appetite-improving effect of orally administered THM in treating anorexia in patients with cancer. It adhered to rigorous methodologies such as the Preferred Reporting Items for Systematic Reviews and Meta-Analysis and the Cochrane handbook, and addressed the quality of evidence using the GRADE profile. Furthermore, the protocol of this study was registered with the International Prospective Register of Systematic Review, and a search was carried out in various databases without publication language or country restrictions.

## 5 Conclusion

Moderate to low quality of evidence suggests that THM is more effective than appetite stimulants or usual care in improving anorexia in patients with cancer. The administration of THM was not related to serious adverse events. However, large-sample, double-blind RCTs with rigorous methodological evidence are required to fully define the efficacy and safety of THM in treating cancer-related anorexia. Future studies with a generalized population, standardized herbal ingredients, and an appropriate follow-up duration are recommended.

## Data Availability

The original contributions presented in the study are included in the article/Supplementary Material, further inquiries can be directed to the corresponding author.
