# Supplementary material for: Traditional herbal medicine for anorexia in patients with cancer: a systematic review and meta-analysis of randomized controlled trials
Source: Front Pharmacol. 2023 Jun 27;14:1203137. doi: 10.3389/fphar.2023.1203137 (PMC10333490; doi:10.3389/fphar.2023.1203137)
Supplement: Supplementary file 1 [file Table1.DOCX]

Supplementary Material

Traditional Herbal Medicine for Anorexia in Patients with Cancer: A Systematic Review and Meta-Analysis of Randomized Controlled Trials

**Su Bin Park, Jee-Hyun Yoon, Eun Hye Kim, Hayun Jin, Seong Woo Yoon^*^**

*** Correspondence:** Seong Woo Yoon: stepano212@hanmail.net

# Supplementary Material S1. Search terms for each database

## PubMed

#1 Search "Neoplasms"[Mesh]

#2 Search (neoplasm* or cancer* or carcino* or malignan* or tumor* or tumour*)

#3 Search ("Neoplasms"[Mesh]) OR ((neoplasm* or cancer* or carcino* or malignan* or tumor* or tumour*))

#4 Search "Anorexia"[Mesh]

#5 Search "Cachexia"[Mesh]

#6 Search Anorexia[tiab]

#7 Search #4 OR #5 OR #6

#8 Search “Drugs, Chinese Herbal”[MeSH] OR "Plants, Medicinal”[MeSH] OR “Herbal Medicine”[MeSH] OR “Medicine, Kampo”[MeSH] OR "Medicine, Korean Traditional”[MeSH] OR "Medicine, Chinese Traditional”[MeSH]

#9 Search “traditional Korean medicine”[tiab] OR “traditional Chinese medicine”[tiab] OR “traditional oriental medicine”[tiab] OR “Kampo medicine”[tiab] OR herb*[tiab] OR decoction*[tiab] OR botanic*[tiab]

#10 Search juzentaihoto [Supplementary Concept]

#11 Search "liu-jun-zi-tang" [Supplementary Concept]

#12 Search #8 OR #9 OR #10 OR #11

#13 Search #3 AND #7 AND #12

## Cochrane Library

#1 MeSH descriptor: [Neoplasms] explode all trees

#2 neoplasm* or cancer* or carcino* or malignan* or tumor* or tumour*

#3 #1 OR #2

#4 MeSH descriptor: [Anorexia] explode all trees

#5 MeSH descriptor: [Cachexia] explode all trees

#6 #4 OR #5

#7 MeSH descriptor: [Medicine, Kampo] explode all trees

#8 MeSH descriptor: [Medicine, Korean Traditional] explode all trees

#9 MeSH descriptor: [Medicine, Chinese Traditional] explode all trees

#10 #7 OR #8 OR #9

#11 #3 AND #6 AND #10

## EMBASE

#1 'neoplasms'/exp

#2 neoplasm* OR cancer* OR carcino* OR malignan* OR tumor* OR tumour*

#3 #1 OR #2

#4 'anorexia'/exp

#5 'cachexia'/exp

#6 #4 OR #5

#7 'Chinese medicine'/exp

#8 'Korean medicine'/exp

#9 'Kampo medicine'/exp

#10 'juzentaihoto'/exp

#11 #7 OR #8 OR #9 OR #10

#12 #3 AND #6 AND #11

#13 [cochrane review]/lim OR [systematic review]/lim OR [controlled clinical trial]/lim OR [randomized controlled trial]/lim OR [meta analysis]/lim

#14 #12 AND #13

## CNKI

#1 癌症

#2 肿瘤

#3 化疗

#4 OR / 1-3

#5 食欲不振

#6 厌食

#7 食欲减退

#8 恶病质

#9 OR / 5-8

#10 #4 AND #9

## CiNii

#1 (Cancer OR neoplasms) AND (anorexia OR cachexia) AND (rikkunshito OR juzen-taiho-to OR Kampo)

## JSOM

#1 Cancer AND anorexia

## KMBASE

#1 ([KEYWORD=cancer] OR [KEYWORD=neoplasms])

#2 ([ABSTRACT=anorexia] OR [ABSTRACT=cachexia])

#3 (([ABSTRACT=traditional medicine] OR [ABSTRACT=korean medicine]) OR [ABSTRACT=herb])

#4 ((([KEYWORD=cancer] OR [KEYWORD=neoplasms]) AND (([ABSTRACT=anorexia] OR [ABSTRACT=cachexia])) AND (([ABSTRACT=traditional medicine] OR [ABSTRACT=korean medicine]) OR [ABSTRACT=herb]))

## KISS

#1 cancer AND anorexia AND Korean medicine

#2 cancer AND anorexia AND traditional medicine

#3 OR / 1-2

## NDSL

#1 ABSTRACT=cancer

#2 ABSTRACT=anorexia | cachexia

#3 ABSTRACT=Korean medicine | traditional medicine | herb

#4 AND / 1-3

## OASIS

#1 cancer AND anorexia AND Korean medicine

#2 cancer AND fatigue AND traditional medicine

#3 cancer AND fatigue AND herb

#4 OR / 1-3
