# Supplementary material for: Traditional herbal medicine for anorexia in patients with cancer: a systematic review and meta-analysis of randomized controlled trials
Source: Front Pharmacol. 2023 Jun 27;14:1203137. doi: 10.3389/fphar.2023.1203137 (PMC10333490; doi:10.3389/fphar.2023.1203137)
Supplement: Supplementary file 2 [file Table2.DOCX]

Supplementary Material

Traditional Herbal Medicine for Anorexia in Patients with Cancer: A Systematic Review and Meta-Analysis of Randomized Controlled Trials

**Su Bin Park, Jee-Hyun Yoon, Eun Hye Kim, Hayun Jin, Seong Woo Yoon^*^**

*** Correspondence:** Seong Woo Yoon: stepano212@hanmail.net

# Supplementary Material S2. Herbal formulas and its components in the included studies

| Study | Name of the prescription | Composition |
| --- | --- | --- |
| Cui et al. (2015) | Kaiwei Jinshi Tang | Root of *Codonopsis pilosula* (Franch.) Nannf. 15 g,  Rhizome of *Atractylodes macrocephala* Koidz 15 g,  Sclerotium of *Poria cocos* (Schw.) Wolf 15 g,  Rind of *Citrus aurantium* L. 6 g,  Tuber of Pinellia ternata (Thunb.) Makino 10 g,  Flower of *Syzygium aromaticum* (L.) Merr. & L. M. Perry 3 g,  Root of *Dolomiaea costus* (Falc.) Kasana & A. K. Pandey 10 g,  Leaves and stems of *Agastache rugosa* (Fisch. & C. A. Mey.) Kuntze 15 g,  Seed of *Nelumbo nucifera* Gaertn. 15 g,  Bark of *Magnolia officinalis* Rehder & E.H.Wilson 10 g,  Fruit of *Wurfbainia villosa* (Lour.) Skornick. & A. D. Poulsen 6 g,  Fruit of *Hordeum vulgare* L. 15 g,  Massa Medicata Fermentata 15 g,  Prepared root and rhizome of *Glycyrrhiza uralensis* Fisch. ex DC. 6 g |
| Hu et al. (2007) | Shenqijiaocao Decoction | Root of *Glehnia littoralis* (A. Gray) F.Schmidt ex Miq.,  Root of *Astragalus mongholicus* Bunge,  Asini Corii Colla,  Herb of *Agrimonia pilosa* Ledeb.,  Herb of *Scleromitrion diffusum* (Willd.) R.J.Wang,  Rhizome of *Dioscorea oppositifolia* L.,  Sclerotium of *Poria cocos* (Schw.) Wolf,  Carapace of *Chinemys reevesii* (Gray),  Rhizome of *Polygonatum sibiricum* Redouté,  Rhizome of *Curcuma phaeocaulis* Valeton,  Rind of *Citrus aurantium* L.,  Tuber of *Arisaema heterophyllum* Blum |
| Huang et al. (2015) | Xiangsha Liujunzi decoction | Root of *Codonopsis pilosula* (Franch.) Nannf. 30 g,  Sclerotium of *Poria cocos* (Schw.) Wolf 25 g,  Rhizome of *Atractylodes macrocephala* Koidz 15 g,  Rind of *Citrus aurantium* L. 15 g,  Tuber of Pinellia ternata (Thunb.) Makino 12 g,  Root of *Dolomiaea costus* (Falc.) Kasana & A. K. Pandey 15 g,  Fruit of *Wurfbainia villosa* (Lour.) Skornick. & A. D. Poulsen 15 g,  Fruit of *Citrus aurantium* L. 15 g,  Fruit of *Hordeum vulgare* L. 30 g,  Membrane of *Gallus gallus domesticus* Brisson 15 g,  Fruit of *Oryza sativa* L. 30 g,  Root and rhizome of *Glycyrrhiza uralensis* Fisch. ex DC. 10 g |
| Kuang et al. (2009) | Yiqi Yangyin Decoction | Root of *Pseudostellaria heterophylla* (Miq.) Pax 15 g,  Root of *Glehnia littoralis* (A. Gray) F. Schmidt ex Miq. 15 g,  Root tuber of *Ophiopogon japonicus* (Thunb.) Ker Gawl. 15 g,  Rhizome of *Dioscorea oppositifolia* L. 15 g,  Rhizome of *Polygonatum sibiricum* Redouté 15 g,  Stem of *Dendrobium nobile* Lindl. 30 g,  Kernel of *Coix lacryma-jobi var. ma-yuen* (Rom. Caill.) Stapf 30 g,  Herb of *Scleromitrion diffusum* (Willd.) R. J. Wang 30 g,  Rhizome of *Atractylodes macrocephala* Koidz 10 g,  Root of *Astragalus mongholicus* Bunge 10 g,  Sclerotium of *Poria cocos* (Schw.) Wolf 10 g,  Carapace of *Chinemys reevesii* (Gray) 10 g,  Herb of *Agrimonia pilosa* Ledeb. 20 g,  Root and rhizome of *Glycyrrhiza uralensis* Fisch. ex DC. 6 g |
| Li et al. (2011) | Jianpi Huatan Decoction | Root of *Astragalus mongholicus* Bunge 30 g,  Root of *Pseudostellaria heterophylla* (Miq.) Pax 15 g,  Rhizome of *Atractylodes macrocephala* Koidz 10 g,  Kernel of *Coix lacryma-jobi var. ma-yuen* (Rom. Caill.) Stapf 30 g,  Rind of *Citrus aurantium* L. 10 g,  Shell of *Ostrea gigas Thunberg* 30 g,  Pheretima 15 g,  Tuber of Pinellia ternata (Thunb.) Makino 12 g,  Rhizome of *Smilax china* L. 30 g,  Fruit of *Crataegus pinnatifida* Bunge 10 g,  Fruit of *Hordeum vulgare* L. 10 g,  Massa Medicata Fermentata 10 g,  Root and rhizome of *Glycyrrhiza uralensis* Fisch. ex DC. 10 g |
| Li et al. (2014) | Jiaweizhizhu particles | Fruit of *Citrus aurantium* L. 20 g,  Rhizome of *Atractylodes macrocephala* Koidz 20 g,  Rind of *Citrus aurantium* L. 10 g,  Tuber of Pinellia ternata (Thunb.) Makino 10 g,  Massa Medicata Fermentata 10 g,  Fruit of *Hordeum vulgare* L. 10 g,  Fruit of *Crataegus pinnatifida* Bunge 10 g,  Leaf of *Nelumbo nucifera* Gaertn. 6 g |
| Wang et al. (2013) | Shuyu Pill | Rhizome of *Dioscorea oppositifolia* L. 30 g,  Root of *Angelica sinensis* (Oliv.) Diels 15 g,  Twig of *Neolitsea cassia* (L.) Kosterm. 6 g,  Prepared root of *Rehmannia glutinosa* (Gaertn.) DC. 20 g,  Root of *Codonopsis pilosula* (Franch.) Nannf. 10 g,  Rhizome of *Conioselinum anthriscoides “Chuanxiong”* 10 g,  Root of *Paeonia lactiflora* Pall. 15 g,  Rhizome of *Atractylodes macrocephala* Koidz 15 g,  Root tuber of *Ophiopogon japonicus* (Thunb.) Ker Gawl. 10 g,  Sclerotium of *Poria cocos* (Schw.) Wolf 20 g,  Root of *Platycodon grandiflorus* (Jacq.) A. DC. 10 g,  Seed of *Prunus armeniaca* L. 10 g,  Fruit of *Citrus aurantium* L. 10 g,  Root of *Bupleurum chinense* DC. 10 g,  Asini Corii Colla 10 g,  Fruit of *Hordeum vulgare* L. 30 g,  Membrane of *Gallus gallus domesticus* Brisson 10 g,  Root and rhizome of *Glycyrrhiza uralensis* Fisch. ex DC. 6 g |
| Wang et al. (2018) | modified Xiangshaliujun Decoction | Root of *Codonopsis pilosula* (Franch.) Nannf. 15 g,  Rhizome of *Atractylodes macrocephala* Koidz 10 g,  Sclerotium of *Poria cocos* (Schw.) Wolf 10 g,  Rind of *Citrus aurantium* L. 10 g,  Tuber of Pinellia ternata (Thunb.) Makino 10 g,  Root and rhizome of *Glycyrrhiza uralensis* Fisch. ex DC. 6 g,  Root of *Dolomiaea costus* (Falc.) Kasana & A. K. Pandey 6 g,  Fruit of *Wurfbainia villosa* (Lour.) Skornick. & A. D. Poulsen 3 g |
| Yu (2015) | Jianpi Huatan Decoction | Root of *Astragalus mongholicus* Bunge 30 g,  Kernel of *Coix lacryma-jobi var. ma-yuen* (Rom. Caill.) Stapf 30 g,  Shell of *Ostrea gigas Thunberg* 30 g,  Rhizome of *Smilax china* L. 30 g,  Root of *Pseudostellaria heterophylla* (Miq.) Pax 15 g,  Pheretima 15 g,  Tuber of Pinellia ternata (Thunb.) Makino 12 g,  Rind of *Citrus aurantium* L. 10 g,  Root and rhizome of *Glycyrrhiza uralensis* Fisch. ex DC. 10 g,  Fruit of *Crataegus pinnatifida* Bunge 10 g,  Fruit of *Hordeum vulgare* L. 10 g,  Massa Medicata Fermentata 10 g |
| Zhang (2000) | Traditional herbal medicine | Root of *Astragalus mongholicus* Bunge 30 g,  Stem of *Spatholobus suberectus* Dunn 30 g,  Kernel of *Coix lacryma-jobi var. ma-yuen* (Rom. Caill.) Stapf 30 g,  Root of *Codonopsis pilosula* (Franch.) Nannf. 12 g,  Root of *Angelica sinensis* (Oliv.) Diels 12 g,  Sclerotium of *Poria cocos* (Schw.) Wolf 12 g,  Root of *Paeonia lactiflora* Pall. 12 g,  Rhizome of *Dioscorea oppositifolia* L. 12 g,  Fruit of *Lycium barbarum* L. 12 g,  Rind of *Citrus aurantium* L. 12 g,  Massa Medicata Fermentata 12 g,  Fruit of *Hordeum vulgare* L. 12 g,  Fruit of *Crataegus pinnatifida* Bunge 12 g |
| Yi (2016) | Kaiwei Jinshi Tang | Root of *Codonopsis pilosula* (Franch.) Nannf. 12 g,  Rind of *Citrus aurantium* L. 6 g,  Flower of *Syzygium aromaticum* (L.) Merr. & L.M.Perry 2 g,  Sclerotium of *Poria cocos* (Schw.) Wolf 12 g,  Fruit of *Hordeum vulgare* L. 12 g,  Tuber of Pinellia ternata (Thunb.) Makino 8 g,  Fruit of *Wurfbainia villosa* (Lour.) Skornick. & A. D. Poulsen 5 g,  Leaves and stems of *Agastache rugosa* (Fisch. & C. A. Mey.) Kuntze 12 g,  Bark of *Magnolia officinalis* Rehder & E. H. Wilson 8 g,  Root and rhizome of *Glycyrrhiza uralensis* Fisch. ex DC. 5 g,  Massa Medicata Fermentata 12 g,  Seed of *Nelumbo nucifera* Gaertn. 12 g |
| Zhang et al. (2014) | Xiaoyan Decoction | Root of *Astragalus mongholicus* Bunge,  Root of *Pseudostellaria heterophylla* (Miq.) Pax,  Fruit spike of *Prunella vulgaris* L.,  Rhizome of *Curcuma longa* L.,  Herb of *Scleromitrion diffusum* (Willd.) R. J. Wang |
| Zhang et al. (2020) | Fuzi Lizhong Decoction | Prepared daughter root of *Aconitum carmichaeli* Debeaux 15 g,  Root of *Codonopsis pilosula* (Franch.) Nannf. 30 g,  Rhizome of *Atractylodes macrocephala* Koidz 30 g,  Dried rhizome of *Zingiber officinale* Roscoe 15 g,  Root and rhizome of *Glycyrrhiza uralensis* Fisch. ex DC. 15 g,  Fruit of *Wurfbainia villosa* (Lour.) Skornick. & A. D. Poulsen 10 g,  Fruit of *Hordeum vulgare* L. 30 g,  Massa Medicata Fermentata 20 g,  Fruit of *Crataegus pinnatifida* Bunge 15 g |
| Cai et al. (2003) | Buzhong Yiqi Tang | Root of *Codonopsis pilosula* (Franch.) Nannf. 15 g,  Sclerotium of *Poria cocos* (Schw.) Wolf 15 g,  Rhizome of *Atractylodes macrocephala* Koidz. 15 g,  Root of *Angelica sinensis* (Oliv.) Diels 15 g,  Fruit of *Setaria italica* (L.*) P. Beauv.* 15 g,  Fruit of *Hordeum vulgare* L. 15 g,  Root of *Astragalus mongholicus* Bunge 30 g,  Rhizome of *Actaea cimicifuga* L. 9 g,  Root of *Bupleurum chinense* DC. 9 g,  Fruit of *Wurfbainia villosa* (Lour.) Skornick. & A. D. Poulsen 6 g,  Root and rhizome of *Glycyrrhiza uralensis* Fisch. ex DC. 6 g |
| Chen (2007) | Zhipu Liujunzi Decoction | Fruit of *Citrus aurantium* L. 10 g,  Bark of *Magnolia officinalis* Rehder & E. H. Wilson 10 g,  Tuber of *Pinellia ternata* (Thunb.) Makino 10 g,  Root and rhizome of *Glycyrrhiza uralensis* Fisch. ex DC. 10 g,  Membrane of *Gallus gallus domesticus* Brisson 10 g,  Root of *Codonopsis pilosula* (Franch.) Nannf. 30 g,  Sclerotium of *Poria cocos* (Schw.) Wolf 30 g,  Rhizome of *Atractylodes macrocephala* Koidz 15 g,  Rind of *Citrus aurantium* L*.* 12 g,  Fruit of *Crataegus pinnatifida* Bunge 12 g,  Fruit of *Hordeum vulgare* L. 12 g,  Massa Medicata Fermentata 12 g |
| Deng (1997) | Xiangsha Erya Kaiwei Decoction | Root of *Codonopsis pilosula* (Franch.) Nannf. 30 g,  Fruit of *Hordeum vulgare* L. 30 g,  Fruit of *Setaria italica* (L*.) P. Beauv.* 30 g,  Root of *Dolomiaea costus* (Falc.) Kasana & A. K. Pandey 10 g,  Fruit of *Wurfbainia villosa* (Lour.) Skornick. & A. D. Poulsen 15 g,  Tuber of Pinellia ternata (Thunb.) Makino 15 g,  Sclerotium of *Poria cocos* (Schw.) Wolf 25 g,  Seed of *Lablab purpureus subsp. purpureus* 20 g |
| Hamai et al. (2019) | Rikkunshito | Rhizome of *Atractylodes lancea* (Thunb.) DC. 4 g,  Root of *Panax ginseng* C. A. Mey. 4 g,  Tuber of *Pinellia ternata* (Thunb.) Makino 4 g,  Sclerotium of *Poria cocos* (Schw.) Wolf 4 g,  Fruit of *Ziziphus jujuba* Mill. 2 g,  Pericarp of *Citrus aurantium* L. 2 g,  Root and stolon of *Glycyrrhiza uralensis* Fisch. ex DC. 1 g,  Rhizome of *Zingiber officinale* Roscoe 0.5 g |
| He et al. (2007) | Bazhen granule | Root of *Paeonia lactiflora* Pall.,  Rhizome of *Atractylodes macrocephala* Koidz,  Rhizome of *Conioselinum anthriscoides “Chuanxiong”,*  Root of *Angelica sinensis* (Oliv.) Diels,  Root of *Codonopsis pilosula* (Franch.) Nannf.,  Sclerotium of *Poria cocos* (Schw.) Wolf,  Root and rhizome of *Glycyrrhiza uralensis* Fisch. ex DC.,  Prepared root of *Rehmannia glutinosa* (Gaertn.) DC. |
| Ohnishi et al. (2017) | Rikkunshito | Rhizome of *Atractylodes lancea* (Thunb.) DC.,  Root of *Panax ginseng* C. A. Mey.,  Tuber of *Pinellia ternata* (Thunb.) Makino,  Sclerotium of *Poria cocos* (Schw.) Wolf,  Fruit of *Ziziphus jujuba* Mill.,  Pericarp of *Citrus aurantium* L.,  Root and stolon of *Glycyrrhiza uralensis* Fisch. ex DC.,  Rhizome of *Zingiber officinale* Roscoe |
| Ohno et al. (2011) | Rikkunshito | Rhizome of *Atractylodes lancea* (Thunb.) DC. 4 g,  Root of *Panax ginseng* C. A. Mey. 4 g,  Tuber of *Pinellia ternata* (Thunb.) Makino 4 g,  Sclerotium of *Poria cocos* (Schw.) Wolf 4 g,  Fruit of *Ziziphus jujuba* Mill. 2 g,  Pericarp of *Citrus aurantium* L. 2 g,  Root and stolon of *Glycyrrhiza uralensis* Fisch. ex DC. 1 g,  Rhizome of *Zingiber officinale* Roscoe 0.5 g, |
| Qiu et al. (2013) | Shenling Baizhu san | Root of *Codonopsis pilosula* (Franch.) Nannf. 15 g,  Rhizome of *Atractylodes macrocephala* Koidz 20 g,  Sclerotium of *Poria cocos* (Schw.) Wolf 20 g,  Seed of *Nelumbo nucifera* Gaertn. 30 g,  Kernel of *Coix lacryma-jobi var. ma-yuen* (Rom. Caill.) Stapf 30 g,  Fruit of *Wurfbainia villosa* (Lour.) Skornick. & A. D. Poulsen 9 g,  Seed of *Lablab purpureus subsp. purpureus* 30 g,  Rhizome of *Dioscorea oppositifolia* L. 30 g,  Rind of *Citrus aurantium*L. 10 g,  Root of *Platycodon grandiflorus* (Jacq.) A. DC. 10 g,  Root and rhizome of *Glycyrrhiza uralensis* Fisch. ex DC. 10 g,  Fruit of *Hordeum vulgare* L. 30 g,  Fruit of *Setaria italica (*L.*) P. Beauv.* 30 g, |
| Wang et al. (2013a) | Shuyu Pill | Rhizome of *Dioscorea oppositifolia* L. 30 g,  Root of *Angelica sinensis* (Oliv.) Diels 15 g,  Twig of *Neolitsea cassia* (L.) Kosterm. 6 g,  Prepared root of *Rehmannia glutinosa* (Gaertn.) DC. 20 g,  Root of *Codonopsis pilosula* (Franch.) Nannf. 10 g,  Rhizome of *Conioselinum anthriscoides “Chuanxiong”* 10 g,  Root of *Paeonia lactiflora* Pall. 15 g,  Rhizome of *Atractylodes macrocephala* Koidz 15 g,  Root tuber of *Ophiopogon japonicus* (Thunb.) Ker Gawl. 10 g,  Sclerotium of *Poria cocos* (Schw.) Wolf 20 g,  Root of *Platycodon grandiflorus* (Jacq.) A. DC. 10 g,  Seed of *Prunus armeniaca* L. 10 g,  Fruit of *Citrus aurantium* L. 10 g,  Root of *Bupleurum chinense* DC. 10 g,  Asini Corii Colla 10 g,  Fruit of *Hordeum vulgare* L. 30 g,  Membrane of *Gallus gallus domesticus* Brisson 10 g,  Prepared root and rhizome of *Glycyrrhiza uralensis* Fisch. ex DC. 6 g |
| Yan et al. (2012) | Xiangsha Zhizhu Jiawe Decoction | Rhizome of *Cyperus rotundus* L. 15 g,  Fruit of *Wurfbainia villosa* (Lour.) Skornick. & A. D. Poulsen 15 g,  Rhizome of *Atractylodes macrocephala* Koidz 30 g,  Fruit of *Citrus aurantium* L. 10 g,  Massa Medicata Fermentata 30 g,  Fruit of *Hordeum vulgare* L. 30 g,  Fruit of *Crataegus pinnatifida* Bunge 15 g,  Leaf of *Nelumbo nucifera* Gaertn. 6 g |
| Yoshiya et al. (2020) | Rikkunshito | Rhizome of *Atractylodes lancea* (Thunb.) DC. 4 g,  Root of *Panax ginseng* C. A. Mey. 4 g,  Tuber of *Pinellia ternata* (Thunb.) Makino 4 g,  Sclerotium of *Poria cocos* (Schw.) Wolf 4 g,  Fruit of *Ziziphus jujuba* Mill. 2 g,  Pericarp of *Citrus aurantium* L. 2 g,  Root and stolon of *Glycyrrhiza uralensis* Fisch. ex DC. 1 g,  Rhizome of *Zingiber officinale* Roscoe 0.5 g |
| Ko et al. (2021) | Rikkunshito | Rhizome of *Atractylodes lancea* (Thunb.) DC. 4 g,  Root of *Panax ginseng* C. A. Mey. 4 g,  Tuber of *Pinellia ternata* (Thunb.) Makino 4 g,  Sclerotium of *Poria cocos* (Schw.) Wolf 4 g,  Fruit of *Ziziphus jujuba* Mill. 2 g,  Pericarp of *Citrus aurantium* L. 2 g,  Root and stolon of *Glycyrrhiza uralensis* Fisch. ex DC. 1 g,  Rhizome of *Zingiber officinale* Roscoe 0.5 g |
| Cheon et al. (2017) | Sipjeondaebo-Tang | Bark of *Neolitsea cassia* (L.) Kosterm. 1 g,  Root of *Paeonia lactiflora* Pall. 1 g,  Rhizome of *Atractylodes lancea* (Thunb.) DC. 1 g,  Root of *Panax ginseng* C. A. Mey. 1 g,  Rhizome of *Ligusticum officinale* (Makino) Kitag. 1 g,  Root of *Astragalus mongholicus* Bunge 1 g,  Sclerotium of *Poria cocos* (Schw.) Wolf 1 g,  Prepared root of *Rehmannia glutinosa* (Gaertn.) DC. 1 g,  Root of *Angelica gigas* Nakai 1 g,  Root and rhizome of *Glycyrrhiza uralensis* Fisch. ex DC. 0.5 g |
