# Supplementary material for: Traditional herbal medicine for anorexia in patients with cancer: a systematic review and meta-analysis of randomized controlled trials
Source: Front Pharmacol. 2023 Jun 27;14:1203137. doi: 10.3389/fphar.2023.1203137 (PMC10333490; doi:10.3389/fphar.2023.1203137)
Supplement: Supplementary file 3 [file Table3.DOCX]

Supplementary Material

Traditional Herbal Medicine for Anorexia in Patients with Cancer: A Systematic Review and Meta-Analysis of Randomized Controlled Trials

**Su Bin Park, Jee-Hyun Yoon, Eun Hye Kim, Hayun Jin, Seong Woo Yoon^*^**

*** Correspondence:** Seong Woo Yoon: stepano212@hanmail.net

# Supplementary Material S3. The frequency of herbs in the included studies

| Frequency of herbs | Scientific name |
| --- | --- |
| 22 | *Citrus aurantium* L. |
| 20 | *Glycyrrhiza uralensis* Fisch. ex DC. |
| 20 | *Poria cocos* (Schw.) Wolf |
| 15 | *Hordeum vulgare* L |
| 14 | *Atractylodes macrocephala* Koidz |
| 14 | *Pinellia ternata* (Thunb.) Makino |
| 13 | *Codonopsis pilosula* (Franch.) Nannf. |
| 9 | Massa Medicata Fermentata |
| 9 | *Wurfbainia villosa* (Lour.) Skornick. & A. D. Poulsen |
| 8 | *Astragalus mongholicus* Bunge |
| 7 | *Crataegus pinnatifida* Bunge |
| 6 | *Atractylodes lancea* (Thunb.) DC. |
| 6 | *Dioscorea oppositifolia* L. |
| 6 | *Panax ginseng* C. A. Mey. |
| 6 | *Zingiber officinale* Roscoe |
| 5 | *Angelica sinensis* (Oliv.) Diels |
| 5 | *Coix lacryma-jobi var. ma-yuen* (Rom. Caill.) Stapf |
| 5 | *Nelumbo nucifera* Gaertn. |
| 5 | *Paeonia lactiflora* Pall. |
| 5 | *Ziziphus jujuba* Mill. |
| 4 | *Dolomiaea costus* (Falc.) Kasana & A. K. Pandey |
| 4 | *Gallus gallus domesticus* Brisson |
| 4 | *Pseudostellaria heterophylla* (Miq.) Pax |
| 4 | *Rehmannia glutinosa* (Gaertn.) DC. |
| 3 | Asini Corii Colla |
| 3 | *Bupleurum chinense* DC. |
| 3 | *Conioselinum anthriscoides “Chuanxiong”* |
| 3 | *Magnolia officinalis* Rehder & E.H.Wilson |
| 3 | *Neolitsea cassia* (L.) Kosterm. |
| 3 | *Ophiopogon japonicus* (Thunb.) Ker Gawl. |
| 3 | *Platycodon grandiflorus* (Jacq.) A.DC. |
| 3 | *Scleromitrion diffusum* (Willd.) R.J.Wang |
| 3 | *Setaria italica* (L.*) P.Beauv.* |
| 2 | *Agastache rugosa* (Fisch. & C. A. Mey.) Kuntze |
| 2 | *Agrimonia pilosa* Ledeb. |
| 2 | *Chinemys reevesii* (Gray) |
| 2 | *Glehnia littoralis* (A. Gray) F. Schmidt ex Miq. |
| 2 | *Lablab purpureus subsp. purpureus* |
| 2 | *Ostrea gigas Thunberg* |
| 2 | Pheretima |
| 2 | *Polygonatum sibiricum* Redouté |
| 2 | *Prunus armeniaca* L. |
| 2 | *Smilax china* L. |
| 2 | *Syzygium aromaticum* (L.) Merr. & L. M. Perry |
| 1 | *Aconitum carmichaeli* Debeaux |
| 1 | *Actaea cimicifuga* L. |
| 1 | *Angelica gigas* Nakai Blum |
| 1 | *Arisaema heterophyllum* |
| 1 | *Curcuma longa* L. |
| 1 | *Curcuma phaeocaulis* Valeton |
| 1 | *Cyperus rotundus* L. |
| 1 | *Dendrobium nobile* Lindl. |
| 1 | *Ligusticum officinale* (Makino) Kitag. |
| 1 | *Lycium barbarum* L. |
| 1 | *Oryza sativa* L. |
| 1 | *Prunella vulgaris* L. |
| 1 | *Spatholobus suberectus* Dunn |
