# Supplementary material for: Traditional herbal medicine for anorexia in patients with cancer: a systematic review and meta-analysis of randomized controlled trials
Source: Front Pharmacol. 2023 Jun 27;14:1203137. doi: 10.3389/fphar.2023.1203137 (PMC10333490; doi:10.3389/fphar.2023.1203137)
Supplement: Supplementary file 4 [file Table4.DOCX]

Supplementary Material

Traditional Herbal Medicine for Anorexia in Patients with Cancer: A Systematic Review and Meta-Analysis of Randomized Controlled Trials

**Su Bin Park, Jee-Hyun Yoon, Eun Hye Kim, Hayun Jin, Seong Woo Yoon^*^**

*** Correspondence:** Seong Woo Yoon: [stepano212@hanmail.net](mailto:stepano212@hanmail.net)

# Supplementary Material S4. The components of YGJT and modified YGJT in the included studies

|  |  |  | YGJT |  |  | Modified YGJT | | |  |
| --- | --- | --- | --- | --- | --- | --- | --- | --- | --- |
| Composition (g/day) | Hamai (2019) | Ko (2021) | Ohnishi (2017) | Ohno (2011) | Yoshiya (2020) | Chen (2007) | Huang (2015) | Wang (2018) | |
| Root of *Panax ginseng* C. A. Mey. | 4 | 4 | 4 | 4 | 4 | - | - | - | |
| Root of *Codonopsis pilosula* (Franch.) Nannf. | - | - | - | - | - | 30 | 30 | 15 | |
| Rhizome of *Atractylodes lancea* (Thunb.) DC. | 4 | 4 | 4 | 4 | 4 | - | - | - | |
| Rhizome of *Atractylodes macrocephala* Koidz | - | - | - | - | - | 15 | 15 | 10 | |
| Sclerotium of *Poria cocos* (Schw.) Wolf | 4 | 4 | 4 | 4 | 4 | 30 | 25 | 10 | |
| Tuber of *Pinellia ternata* (Thunb.) Makino | 4 | 4 | 4 | 4 | 4 | 10 | 12 | 10 | |
| Pericarp of *Citrus aurantium* L. | 2 | 2 | 2 | 2 | 2 | - | - | - | |
| Rind of *Citrus aurantium* L. | - | - | - | - | - | 12 | 15 | 10 | |
| Fruit of *Ziziphus jujuba* Mill. | 2 | 2 | 2 | 2 | 2 | - | - | - | |
| Root and stolon of *Glycyrrhiza uralensis* Fisch. ex DC. | 1 | 1 | 1 | 1 | 1 | - | - | - | |
| Root and rhizome of *Glycyrrhiza uralensis* Fisch. ex DC. | - | - | - | - | - | 10 | 10 | 6 | |
| Rhizome of *Zingiber officinale* Roscoe | 0.5 | 0.5 | 0.5 | 0.5 | 0.5 | - | - | - | |
| Fruit of *Hordeum vulgare* L. | - | - | - | - | - | 12 | 30 | - | |
| Fruit of *Oryza sativa* L. | - | - | - | - | - | - | 30 | - | |
| Root of *Dolomiaea costus* (Falc.) Kasana & A. K. Pandey | - | - | - | - | - | - | 15 | 6 | |
| Fruit of *Wurfbainia villosa* (Lour.) Skornick. & A. D. Poulsen | - | - | - | - | - | - | 15 | 3 | |
| Fruit of *Citrus aurantium* L. | - | - | - | - | - | 10 | 15 | - | |
| Bark of *Magnolia officinalis* Rehder & E. H. Wilson | - | - | - | - | - | 10 | - | - | |
| Membrane of *Gallus gallus domesticus* Brisson | - | - | - | - | - | 10 | 15 | - | |
| Fruit of *Crataegus pinnatifida* Bunge | - | - | - | - | - | 12 | - | - | |
| Massa Medicata Fermentata | - | - | - | - | - | 12 | - | - | |

YGJT, Yukgunja-Tang
